# A polo-like kinase modulates cytokinesis and flagella biogenesis in *Giardia lamblia*

**DOI:** 10.1186/s13071-021-04687-5

**Published:** 2021-03-31

**Authors:** Eun-Ah Park, Juri Kim, Mee Young Shin, Soon-Jung Park

**Affiliations:** Department of Environmental Medical Biology and Institute of Tropical Medicine, Yonsei University College of Medicine, Seoul, 03722 South Korea

**Keywords:** *Giardia lamblia*, Polo-like kinase, Cell cycle

## Abstract

**Background:**

Polo-like kinases (PLKs) are conserved serine/threonine kinases that regulate the cell cycle. To date, the role of *Giardia lamblia* PLK (GlPLK) in cells has not been studied. Here, we report our investigation on the function of GlPLK to provide insight into the role of this PKL in *Giardia* cell division, especially during cytokinesis and flagella formation.

**Methods:**

To assess the function of GIPLK, *Giardia* trophozoites were treated with the PLK-specific inhibitor GW843286X (GW). Using a putative open reading frame for the PLK identified in the *Giardia* genomic database, we generated a transgenic *Giardia* expressing hemagglutinin (HA)-tagged GlPLK and used this transgenic for immunofluorescence assays (IFAs). GlPLK expression was knocked down using an anti-*glplk* morpholino to observe its effect on the number of nuclei number and length of flagella. *Giardia* cells ectopically expressing truncated GlPLKs, kinase domain + linker (GlPLK-KDL) or polo-box domains (GlPLK-PBD) were constructed for IFAs. Mutant GlPLKs at Lys51, Thr179 and Thr183 were generated by site-directed mutagenesis and then used for the kinase assay. To elucidate the role of phosphorylated GlPLK, the phosphorylation residues were mutated and expressed in *Giardia* trophozoites

**Results:**

After incubating trophozoites with 5 μM GW, the percentage of cells with > 4 nuclei and longer caudal and anterior flagella increased. IFAs indicated that GlPLK was localized to basal bodies and flagella and was present at mitotic spindles in dividing cells. Morpholino-mediated GlPLK knockdown resulted in the same phenotypes as those observed in GW-treated cells*.* In contrast to *Giardia* expressing GlPLK-PBD, *Giardia* expressing GlPLK-KDL was defective in terms of GIPLK localization to mitotic spindles and had altered localization of the basal bodies in dividing cells. Kinase assays using mutant recombinant GlPLKs indicated that mutation at Lys51 or at both Thr179 and Thr183 resulted in loss of kinase activity. *Giardia* expressing these mutant GlPLKs also demonstrated defects in cell growth, cytokinesis and flagella formation.

**Conclusions:**

These data indicate that GlPLK plays a role in *Giardia* cell division, especially during cytokinesis, and that it is also involved in flagella formation.

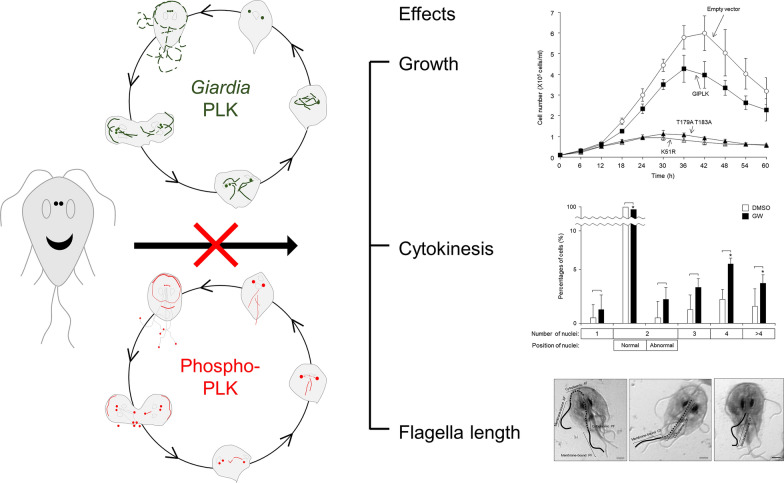

**Supplementary Information:**

The online version contains supplementary material available at 10.1186/s13071-021-04687-5.

## Background

*Giardia lamblia* is a human pathogen that causes diarrheal outbreaks; it is present as either a cyst or a trophozoite. Trophozoites, the multiplying form found in hosts, possess a structure that seems to be bilaterally symmetrical from a side view and exhibits asymmetrical polarity in the anterior/posterior and dorsal/ventral views. These cells have two nuclei and cytoskeletal structures, including an adhesive disc, a median body and four pairs of flagella [1].

Limited information is available on the mechanism responsible for regulating the division of *Giardia* trophozoites. *Giardia lamblia* reportedly has defective cell cycle checkpoints because the cell cycle of *Giardia* trophozoites can progress despite blocked DNA synthesis, double-stranded DNA breaks or defective mitotic spindles [2].* In vitro* cultures of *Giardia* trophozoites are dominated by cells in the gap 2/mitosis (G2/M) phase [3]. Investigations using synchronized cell cultures with chemicals or counterflow centrifugal elutriation have revealed that *Giardia* proteins show phase-specific expression [3–5]. Interestingly, a study using live imaging of *Giardia* cells indicated that cytokinesis occurs 60-fold faster in *Giardia* than in mammalian cells, and that *G. lamblia* uses flagella-mediated membrane tension instead of myosin-dependent contractile rings to initiate daughter cell separation [6].

In mammals, cell division is a complex and well-organized process that incorporates a multitude of protein interactions and macromolecular machinery [7]. This process should be finely and dynamically controlled* via* the actions of interconnected signaling cascades, including aurora kinase (AK), polo-like kinase (PLK) and cyclin-dependent kinase 1 (CDK1) [8]. PLK is a key regulator in this process and has diverged into five paralogues in mammals, including PLK1–5 [9]. In particular, PLK1 is a mitotic kinase with multiple roles in several steps of the cell cycle from G2 to the final step of cytokinesis [10]. These Ser/Thr kinases are defined by the presence of an N-terminal kinase domain (KD) and additional domains, termed polo-box domains (PBDs), which engage in protein interactions [11]. To perform its functions, PLK must be activated and dynamically recruited to distinct subcellular structures spatially and temporally* via* its interaction with the PBD [12].

An investigation of the *Giardia* kinome indicated the presence of an open reading frame (ORF) for PLK, named GL50803_104150 [13]. In the present study, we examined the putative role of PLK using a PLK inhibitor as well as morpholino-mediated knockdown with respect to *G. lamblia* cell division. The autophosphorylation activity of *G. lamblia* PLK (GlPLK) was measured* in vitro*, and its role in cell division was also confirmed* in vivo* using transgenic *G. lamblia* ectopically expressing a mutant GlPLK that lacks critical residue(s) for autophosphorylation.

## Methods

### Culture of *G. lamblia* trophozoites

*Giardia lamblia* trophozoites (strain WB, ATCC30957; American Type Culture Collection, Manassas, VA, USA) were grown in modified TYI-S-33 medium (2% casein digest, 1% yeast extract, 1% glucose, 0.2% NaCl, 0.2% l-cysteine, 0.02% ascorbic acid, 0.2% K_2_HPO_4_, 0.06% KH_2_PO_4_, 10% calf serum and 0.5 mg/mL bovine bile, pH 7.1) at 37 °C [14].

### Scoring of *G. lamblia* cells for cell growth

The 50% inhibitory concentration for cell death (IC_50_) was determined by treating *Giardia* trophozoites (2 × 10^4^ cells/ml) with various concentrations) (5–15 μM) of the PLK inhibitor, GW843682X (GW; Cayman Chemical, Ann Arbor, MI, USA). After treatment for 24 h, the number of parasites per milliliter was determined using a hemocytometer. *Giardia* trophozoites treated with 0.3% dimethyl sulfoxide (DMSO) were used as controls.

Various *Giardia* cells (trophozoites carrying plasmids pKS-3HA.neo, pGlPLK.neo, pGlPLKK51R.neo or pGlPLKT179AT183A.neo) were inoculated into modified TYI-S-33 medium at 1 × 10^4^ cells/ml, and the cell numbers were counted every 6 h for up to 60 h using a hematocytometer.

### Microscopic observation of Giemsa-stained cells

For the microscopic observation of cells, the cells were attached to slides, air-dried and fixed with 100% methanol for 10 min, following which they were stained with 10% Giemsa solution for 40 min and washed with distilled water. After mounting with dibutyl phthalate xylene (Sigma-Aldrich, St. Louis, MO, USA), the slides were observed under an Axiovert 200 microscope (Carl Zeiss AG, Oberkochen, Germany). For each condition, at least 300 cells were examined to determine the number and position of the nuclei. Among cells with two nuclei in normal positions, the number of cells showing nuclei condensation was also recorded. Data are presented as the mean ± standard deviation (SD) of three independent experiments.

To measure flagella length, we observed Giemsa-stained cells under an Axiovert 200 microscope and analyzed their differential interference contrast (DIC) images using Fiji, an open-source platform for biological-image analysis [15]. Each of four types of flagella were divided into cytoplasmic and membrane-bound categories and then measured using the line Freehand Tracing 0mode in ImageJ software (http://imagej.nih.gov/ij/). These data for flagella length were derived from 35 cells per each experiment. Data are presented as the mean ±  SD of three independent experiments.

### Flow cytometry

Both the GW-treated and control *G. lamblia* cells were analyzed for their DNA content using flow cytometry [16]. Briefly, the harvested cells were resuspended in 50 μl TYI-S-33 culture medium and treated with 150 μl of a cell fixative (1% Triton X-100, 40 mM citric acid, 20 mM dibasic sodium phosphate, 200 mM sucrose; pH 3.0) at room temperature for 5 min. The samples were diluted with 350 μl of diluent buffer [125 mM MgCl_2_ in phosphate-buffered saline (PBS: 137 mM NaCl, 2.7 mM KCl, 10.1 mM Na_2_HPO_4_ and 2 mM KH_2_PO_4_, pH 7.4)] and then stored at 4 °C until use. Fixed cells were treated with 2.5 μg RNase A (Sigma-Aldrich) and 10 μg/ml propidium iodide (PI; Sigma-Aldrich) for 30 min at 37 °C. These data obtained* via* flow cytometry were analyzed using FlowJo software version 10.2 (FlowJo Llc, Ashland, OR, USA). Along representative histograms, averages of three independent experiments were presented in bar graphs.

### Construction of *G. lamblia* expressing the hemagglutinin (HA) epitope-tagged GlPLK proteins

A 2184-bp DNA fragment of the *glplk* gene, which comprises the promoter region (150 bp) and the ORF, was amplified from *Giardia* genomic DNA by PCR using two primers, Pplk-F and PLK-PDB-R (Table 1). The NotI and SalI sites were cloned into plasmid pKS-3HA.neo [17] to obtain pGlPLK.neo. The construct was confirmed by DNA sequencing using a sequencing service company (Macrogen, Seoul, Korea).

**Table 1:** Primers and morpholinos used in this study

Name	Nucleotide sequence (5′–3′)^a, b^
Transgenic *Giardia lamblia* expressing HA-tagged GlPLK	
Pplk-F	CATCGCGGCCGCGTAGGCGTCATCCGAGGTGAAC
PLK-NL-R	GTTACGTCGACGTCTATTTGTGGATACTCGGCTT
Pplk-R	GTTACAAGCTTGGGGCTATAAAATTTTACAGAG
PLK-PBD-F	GTTACAAGCTTCCACCGTGCTACATCATGTCCTG
PLK-PBD-R	GTTACGTCGACTCCCCTCCCTGACCGAGCTGCCT
Mopholino sequences	
Control	CCTCTTACCTCAGTTACAATTTATA
Anti-*glplk*	AGCTCCCACCGCAAAAGCCAAAATT
Real-time PCR primers	
PLK-RT-F	GTCACGTTTATGAGCGAGAA
PLK-RT-R	CTATTCCCCTCCCTGACCGA
Actin-F	GTCCGTCATACCATCTGTTC
Actin-R	GTTTCCTCCATACCACACG
Kinase assay	
PLK-GBK-F	GCACGAATTCATGTCCCACAGCAACGCCCCAGAA
PLK-GBK-R	CTACAGCGGCCGCCTATTCCCCTCCCTGACCGAGCT
Mutagenesis of *glplk*	
PLKT183A-F	TGGGCCATGTGTGGA***GCG***CCAAACTTT
PLKT183A-R	GAGAAAGTTTGG***CGC***TCCACACAT
Recombinant protein for antibodies	
rGlPLK-F	GATCGAATTCGATGTCCCACAGCAACGCCCCA
rGlPLK-R	CGATCTCGAGTTCCCCTCCCTGACCGAGCTG
rGlGAP1-F	GATTGAATTCATGCCTATTCGCCTCGGAAT
rGlGAP1-R	GCCTAGCGGCCGCGGCAGCCCTTGGACCCGACGTA
rGlCENH3-F	GATCCATATGATGAGCGGAGGCTCACGG
rGlCENH3-R	TACCGTCGACCCGTAGTGAATTTAAGTT

*glplk Giardia lamblia* polo-like kinase gene, *HA* Hemagglutinin

^a^Restriction enzyme sites are underlined

^b^Mutated bases are indicated as bold and italic letters

An aliquot (20 μg) of pGlPLK.neo was transfected into 1 × 10^7^ *Giardia* trophozoites by electroporation under the following conditions: 350 V, 1000 μF and 700 Ω (Bio-Rad, Hercules, CA, USA). Expression of HA-tagged GlPLK was confirmed by western blotting. *Giardia* trophozoites carrying pKS-3HA.neo were included as empty vectors. For each experiment, five sets of transfection were performed with an 80% success rate. Three of the resulting transfectants were examined for the expression of HA-tagged GlPLK.

Two truncated GlPLK proteins were also ectopically expressed in *Giardia* trophozoites. A 1443-bp DNA fragment encoding the N-terminal portion of GlPLK (KD + linker region) was amplified using the primers Pplk-F and PLK-NL-R (Table 1) and then cloned into the NotI and SalI sites of pKS-3HA.neo to generate pGlPLKKDL.neo. To express the two PBDs of GlPLK, a 150-bp *glplk* promoter region (amplified by PCR using primers Pplk-F and Pplk-R) was cloned into pKS-3HA.neo to produce pPplk-3HA.neo. Subsequently, DNA encoding the PBDs of G1PLK was amplified using the PLK-PBD-F and PLK-PBD-R primers and then cloned into the HindIII and SalI regions of pPplk-3HA.neo to obtain pGlPLKPBD.neo. These plasmids were transfected into *Giardia* trophozoites as described above. The expression of these truncated proteins was examined by western blotting using anti-HA antibodies.

### Western blotting

Extracts prepared from *Giardia* cells carrying plasmids pKS-3HA.neo or pGlPLK.neo were separated via sodium dodecyl sulfate-polyacrylamide gel electrophoresis (SDS-PAGE) and transferred onto a polyvinylidene fluoride membrane (Millipore, Bedford, MA, USA). The membrane was incubated with monoclonal mouse anti-HA antibodies (1:1000; Sigma-Aldrich) in TBST solution (Tris-buffered saline with Tween 20; 50 mM Tris–HCl, 5% skim milk and 0.05% Tween 20) at 4 °C overnight. The membranes were then incubated with horseradish peroxidase-conjugated secondary antibodies, and immunoreactive proteins were visualized using an enhanced chemiluminescence system (Thermo Fisher Scientific, Waltham, MA, USA). Membranes were incubated in a stripping buffer (Thermo Fisher Scientific) at room temperature for 20 min and then reacted with polyclonal rat antibodies against protein disulfide isomerase 1 (PDI1; GL50803_29487) of *G. lamblia* (1:10,000) as the loading control [18].

### Immunofluorescence assay

To obtain more dividing cells for immunofluorescence assays (IFAs), the culture medium of *Giardia* trophozoites grown for 3 days was replaced with fresh medium, and the cells were collected after growing for 3–4 h. *Giardia* cells were attached onto glass slides coated with l-lysine for 10 min and then fixed with chilled methanol for 10 min followed by PBS/0.5% Triton X-100 for 10 min. After blocking for 1 h in PBS/5% goat serum/3% bovine serum albumin, the cells were incubated with primary antibodies overnight at 4 °C and subsequently treated with fluorescent dye-conjugated secondary antibodies. The samples were mounted with ProLong™ Gold Antifade Mountant with DAPI (Molecular Probes, Waltham, MA, USA) and then examined inverted confocal laser scanning microscopy (model LSM700 microscrope; Carl Zeiss AG).

The following antibodies were used at the indicated dilutions: anti-HA mouse monoclonal antibodies (1:100; Sigma-Aldrich), anti-HA rat monoclonal antibodies (1:100; clone 3F10, Roche Applied Science, Mannheim, Germany), anti-acetylated-α-tubulin mouse antibodies (1:800; T7451, clone 6-11B-1, Sigma-Aldrich), anti-GlCentrin rat antibodies (1:100; [18]), Alexa Fluor 488-conjugated goat anti-mouse IgG (1:100; Molecular Probes), Alexa Fluor 488-conjugated goat anti-rat IgG (1:400; Molecular Probes), Alexa Fluor 555-conjugated anti-rat IgG (1:100; Molecular Probes) and Alexa Fluor 568-conjugated anti-mouse IgG (1:100; Molecular Probes).

Antibodies specific to the phosphorylated form of PLK were purchased from Abcam (ab39068; Cambridge, MA, USA) and then used for IFA of *G. lamblia* cells (1:100) along with anti-HA antibodies to discern the localization of phosphorylated GlPLK.

### Morpholino knockdown

Expression of GlPLK was knocked down using a morpholino, as described previously [19]. A specific morpholino for GlPLK was designed by Gene Tools (Philomath, OR, USA) (see Table 1 for sequences). Nonspecific oligomers were used as a control morpholino (Table 1). Cells (5 × 10^6^ in 0.3 ml medium) were treated with the lyophilized morpholino at a final concentration of 100 nM. After electroporation, the cells were grown for various lengths of time, ranging from 12 to 48 h, and analyzed for GlPLK inhibition by western blotting using anti-HA and anti-GlPLK antibodies. The cells at 18 h post-transfection were analyzed for their nuclear phenotypes, including the number and position of nuclei and condensation and flagella length, as described above. In addition, cells transfected with control or anti-*glplk* morpholino were harvested at 6 or 18 h, and then evaluated with respect to their DNA content by flow cytometry followed by analysis using FlowJo software (FlowJo Llc). Transfection was performed at least five times, and the data presented were derived from three independent knockdown experiments.

### Cell cycle synchronization in *G. lamblia* using nocodazole and aphidicolin

*Giardia* trophozoites (5 × 10^5^ cells/ml) were incubated in modified TYI-S-33 medium to 60% confluency. A portion of these cells was treated with 100 nM nocodazole (Sigma-Aldrich) for 2 h and harvested as G2/M-phase cells. The remaining nocodazole-treated cells were treated with 6 μM aphidicolin (Sigma-Aldrich) for 6 h to obtain gap 1/synthesis (G1/S)-phase cells. *Giardia* trophozoites treated with 0.01% DMSO instead of nocodazole and aphidicolin were used as controls.

These cells were then analyzed by flow cytometry to determine the ploidy of their DNA. Intracellular levels of GlPLK protein in the DMSO-treated, nocodazole-treated and nocodazole/aphidicolin-treated *Giardia* cells were determined by western blotting. The intracellular levels of *glplk* transcripts were also measured in these cells.

### Formation of anti-GlPLK, anti-GlGAP and anti-CenH3 antibodies

A 2037-bp *glplk* DNA fragment was amplified by PCR using the primers rGlPLK-F and rGlPLK-R (Table 1), and then cloned into pET32b to produce pET32-GlPLK (Table 2).

**Table 2:** Strains and plasmids used in this study

Organism/plasmid	Description	Source/references
*Giardia lamblia*		
ATCC 30957	Clinical isolate	ATCC
*Escherichia coli*		
DH5α	*supE44*, *ΔlacU169* (*Φ80 lacZ ΔM15*), *hsdR17*, *recA1*, *endA1*, *gyrA96*, *thi-1*, *relA1*	Invitrogen, Thermo Fisher Scientific (Carlsbad, CA, USA)
BL21 (DE3)	*F′*, *ompT*, *hsdSB*(*rB*^*−*^*mB*^*−*^) *gal*, *dcm* (*DE3*)	Invitrogen, Thermo Fisher Scientific
Plasmids		
pKS-3HA.neo	Shuttle vector, Amp^R^, *neo* gene	[17]
pGlPLK.neo	pKS_3HA.neo, 2184 bp, encoding *glplk* (GiardiaDB ID GL50803_104150)	This study
pGlPLKKDL.neo	pKS-3HA.neo, 1443 bp, encoding kinase domain and linker of *glplk*	This study
pPplk-3HA.neo	pKS-3HA.neo, 150 bp, encoding promoter region of *glplk*	This study
pGlPLKPBD.neo	pKS-3HA.neo, 894 bp, encoding promoter region and PBDs of *glplk*	This study
pGlPLKK51R.neo	pKS-3HA.neo, 2184 bp, encoding K51R *glplk*	This study
pGlPLKT179A.neo	pKS-3HA.neo, 2184 bp, encoding T179A *glplk*	This study
pGlPLKT183A.neo	pKS-3HA.neo, 2184 bp, encoding T183A *glplk*	This study
pGlPLKT179AT183A.neo	pKS-3HA.neo, 2184 bp, encoding T179AT183A *glplk*	This study
pGBKT7	Gal4p_(1–147)_ DNA-BD, TRP1, Kan^R^, c-Myc Epitope	Clontech, Takara Bio (Mountainview, CA, USA)
pGBK-GlPLK	pGBKT7, 2037 bp, encoding *glplk*	This study
pGBK-GlPLKK51R	pGBKT7, 2037 bp, encoding K51R *glplk*	This study
pGBK-GlPLKT179A	pGBKT7, 2037 bp, encoding T179A *glplk*	This study
pGBK-GlPLKT183A	pGBKT7, 2037 bp. encoding T183A *glplk*	This study
pGBK-GlPLKT179AT183A	pGBKT7, 2037 bp, encoding T179AT183A *glplk*	This study
pET32b	Expression vector, Amp^R^	Novagen (Merck Biosciences, Merck AG (Darmstadt, Germany)
pET32-GlPLK	pET32b, 2037 bp, encoding GlPLK	This study
pGEX4T-1	Expression vector, Amp^R^, GST	GE Healthcare (Chicago, IL, USA)
pGEX-GlGAP1	pGEX4T-1, 1011 bp, encoding *glgap1*	This study
pET21b	Expression vector, Amp^R^	Novagen
pET-GlCENH3	pET21b, 471 bp, encoding *glcenh3*	This study

*AD* activation domainm *Amp* Ampicillin, *DNA-BD* DNA binding domain, *Kan* kanamycin, *R* resistant

In *Giardia*, a conventional histone 3 and two histone 3 variants, i.e. histone 3 B and centromeric histone 3, have been reported, all of which were found in the nuclei when they are expressed as green fluorescent protein-tagged proteins* in vivo* [20]. One of the histone 3 proteins, i.e. centromeric H3, was expressed as a recombinant protein and then used to make antibodies for a marker for the nuclear protein. A 1011-bp DNA fragment encoding GlGAP1 (GL50803_6687) or a 471-bp DNA fragment encoding GlCenH3 (GL50803_20037) were amplified from the *Giardia* genome. Each fragment was cloned into pGEX4T-1 or pET21b to produce pGEX-GlGAP1 or pET-GlCENH3, respectively (Table 2). Histidine-tagged GlPLK, GST-GlGAP1 and HA-tagged GlCenH3 were overexpressed in *Escherichia coli* BL21 (DE3) with the addition of 1 mM IPTG at 37 °C. The resultant recombinant proteins were excised from the SDS-PAGE gel and used to immunize Sprague–Dawley rats (2 weeks old, female) to produce polyclonal antibodies, as previously described [21]. All primers used are listed in Table 1.

### Quantitative real-time PCR

Total RNA was prepared from interphase, G1/S-phase and G2/M-phase cells using TRIzol (Invitrogen, Thermo Fisher Scientific, Carlsbad, CA, USA) according to the manufacturer’s instructions. A 5-μg aliquot of RNA was converted into complementary DNA (cDNA) using an Improm-II Reverse Transcription System (Promega, Madison, WI, USA). Real-time PCR was performed using a LightCycler System and LightCycler 480 SYBR Green I Master Kit (Roche Applied Science, Penzberg, Germany). The conditions for real-time PCR were as follows: pre-incubation at 95 °C, 5 min; then 94 °C/1 min, 56 °C/1 min, 72 °C/1 min for 45 cycles. The nucleotide sequences of the forward and reverse primers used for real-time PCR are listed in Table 1. The *G. lamblia* actin-related gene (*glactin*; GL50803_15113) transcript was used to normalize the amount of mRNA in the samples, which has been reported to be constitutively expressed during the cell cycle of *G. lamblia* [5]. All experiments were performed with three independently prepared cultures.

### Subcellular protein fractionation

*Giardia lamblia* cells at various cell cycle phases (interphase, G1/S-phase and G2/M-phase cells; 2 × 10^9^ cells) were lysed in hypotonic buffer [10 mM HEPES–KOH, 10 mM KCl, 1.5 mM MgCl_2_, 0.2 mM PEFA1023 (pH 7.9), 0.5% Nonidet P-40 (NP-40), 20 mM NEM, protease inhibitor cocktail], as described previously [22]. After centrifugation for 10 min at 16,000 *g*, supernatants were collected as cytoplasmic extracts. The pellets were treated with high-salt buffer (450 mM NaCl, 50 mM Tris–HCl, 2 mM DTT, 1% NP-40, 20 mM NEM, protease inhibitor cocktail) for 10 min and then centrifuged at 4 °C for 15 min at 16,000 *g*. The supernatants were collected as membrane extracts. Equal amounts of cytoplasmic and membrane extracts were analyzed by western blotting using anti-HA (1:1000), anti-GlGAP1 (Gl50803_6687; 1:10,000), anti-GlCentrin (Gl50803_104685; 1:10,000; [18]) or anti-GlCenH3 antibodies (GL50803_20037; 1:5000).

### *In vitro* transcription/translation synthesis of rGlPLK proteins

The TNT T7 Coupled Reticulocyte Lysate System (Promega) was used for the* in vitro* synthesis of c-Myc-tagged GlPLK. The DNA template (0.5 µg; pGBK-GlPLK, pGBK-GlPLKK51R, pGBK-GlPLKT179A, pGBK-GlPLKT183A and pGBK-GlPLKT179AT183A) was incubated with the transcription/translation mix in a total volume of 50 µl at 30 °C for 90 min. The synthesized protein products were resolved by SDS-PAGE and analyzed by western blotting with mouse anti-c-My antibodies (1:1000; Santa Cruz Biotechnology, Dallas, TX, USA).

### Kinase assay

The rGlPLK proteins, which were prepared as mentioned above, were resuspended in 20 μl kinase buffer (50 mM Tris–HCl, 10% glycerol, 5 mM MgCl_2_, 150 mM NaCl, 50 mM KCl and 1 mM DTT, pH 8.0) and then used for kinase assays in the presence of 2.5 µCi [γ-^32^P]ATP (3000 Ci/mmol; PerkinElmer, Waltham, MA, USA). The kinase reactions were processed for 30 min at 30 °C and then stopped by adding SDS loading buffer. Samples were separated in 12% SDS-PAGE gels, which were then dried and subjected to autoradiography.

### Generation of mutant GlPLK proteins by site-directed mutagenesis

As candidate sites for phosphorylation, Lys51 was modified to Arg, whereas Thr179 and Thr183 were mutated to Ala. The following plasmids were supplied by Macrogen for the* in vitro* synthesis of rGlPLK and the* in vivo* expression of GlPLK in *Giardia*: pGBK-GlPLKK51R, pGBK-GlPLKT179A, pGBK-GlPLKT183A (for* in vitro* synthesis), pGlPLK51R.neo, pGlPLKT179A.neo and pGlPLKT184A.neo (for expression in *Giardia*). Plasmids for mutant rGlPLKT179AT183A were generated by site-directed mutagenesis using primers carrying the substitution. To generate a plasmid for the expression of the T179AT183A double mutant GlPLK in *Giardia*, two DNA fragments were amplified using the pGlPLKT179A.neo plasmid as a template with two primer sets, Pplk-F/PLKT183A-R or PLKT183A-F/PLK-PBD-R. The resulting PCR products were used as templates for a second round of PCR with the primers Pplk-F and PLK-PBD-R. The DNA fragment was then cloned into pKS-3HA.neo, resulting in the pGlPLKT179AT183A.neo plasmid. The plasmid for* in vitro* synthesis of the T179AT183A double mutant rGlPLK was constructed in the same manner. Briefly, two PCR fragments were amplified using PLK-GBK-F/PLKT183A-R or PLKT183A-F/PLK-GBK-R. Using these DNA fragments as templates, a second round of PCR was performed with the primers PLK-GBK-F and PLK-GBK-R to obtain the pGBK-PLKT179AT183A plasmid.

These constructed plasmids for the* in vivo* expression of mutant GlPLKs in *Giardia* were transfected into trophozoites by electroporation and the resulting strains were examined for their nuclear phenotypes and flagella length.

### Statistical analysis

Data are presented as the mean ± SD of three independent experiments. Statistical analyses for pairwise comparisons were performed using Student’s t-test to evaluate the statistical significance of these results. Differences with *P* values of < 0.05 were considered to be significant. Data with *P* values of < 0.01 are indicated with two asterisks, whereas data with *P* values between 0.01 and 0.05 are indicated with a single asterisk.

## Results

### Inhibition of PLK activity affects the cell cycle and flagella biogenesis in *G. lamblia*

In order to define the role of PLK, we treated *G. lamblia* trophozoites with various concentrations of GW843682X (GW), an ATP-competitive inhibitor of PLK1 and PLK3 (Additional file 1: Fig. S1) and observed that the inhibition of growth of *G. lamblia* was inversely related to the GW concentration, with an IC_50_ of 7 μM.

**Fig. 1:**
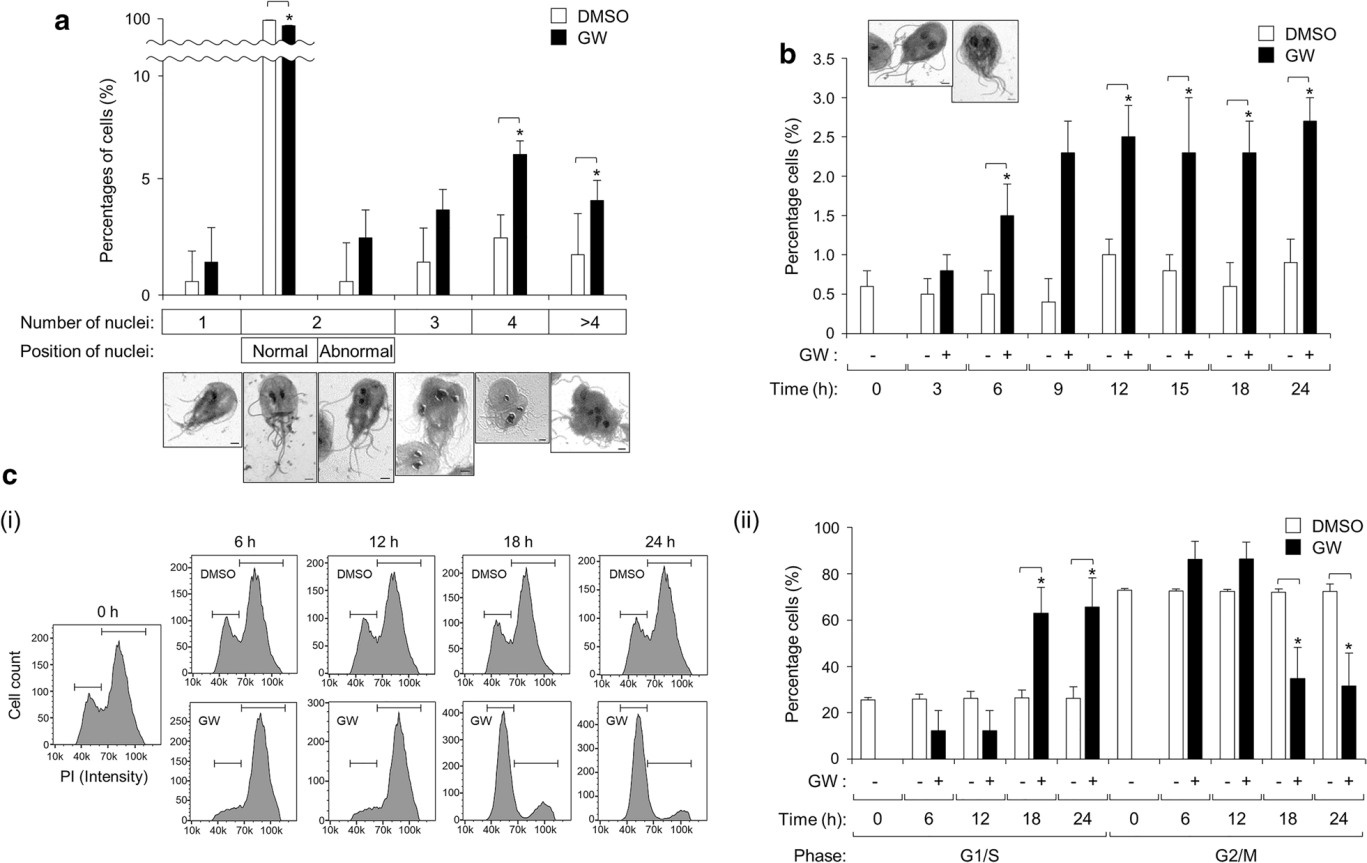
Effects of the polo--like kinase (PLK) inhibitor GW843682X (*GW*) on the nuclear phenotypes of *Giardia lamblia*. **a** Cells were treated with 5 µM GW (closed bars) or 0.1% dimethyl sulfoxide (*DMSO*) (open bars) for 18 h, and then stained with 10% Giemsa solution. At least 300 cells were examined to assess the number and position of the nuclei for each condition under an Axiovert 200 microscope. **b** Cells were treated with 5 µM GW (closed bars) or 0.1% DMSO (open bars) for various lengths of time up to 24 h, and then stained with 10% Giemsa solution. At least 300 cells were examined to record the number of the cells showing nuclear condensation. **c** Cells were treated with 5 µM GW (closed bars) or 0.1% DMSO (open bars) for various lengths of time up to 24 h. After being stained with propidium iodide (*PI*), the ploidy of their DNA was analyzed by flow cytometry. A representative cell for each category is shown. Data are presented as the mean ± standard deviation (SD) of three independent experiments.* G1*/*S* Gap 1/synthesis cell cycle phases,* G2*/*M* gap 2/mitosis cell cycle phases. Asterisk indicates that difference is statistically significant at *P < 0.01. Scale bars: 2 μm

To determine the effect of PLK inhibition on *Giardia* cell division, the cells were treated with 5 µM GW for various lengths of time (range 3–24 h), then stained with Giemsa. The stained cells (> 300 cells for each of the three independent experiments) were scored for the number and position of the nuclei, as follows: cells with one nucleus, with two nuclei in the normal position, with two nuclei in an abnormal position, with three nuclei, with four nuclei and with > 4 nuclei (Additional file 2: Table S1). Based on these data, treatment with 5 µM GW for 18 h was selected to determine the number of nuclei in GW-treated cells and control cells (treated with DMSO for 18 h) (Fig. 1a). A representative cell for each category is shown in Fig. 1a. A majority of the cells were found to have two nuclei in the normal position (97%), and the percentage of these cells decreased to 87%. The number of cells with one nucleus, two abnormally positioned nuclei or three nuclei increased, but this change was not statistically significant. The most notable increase was the percentage of cells with ≥ 4 nuclei, in particular to 5.8 and 2.2% (from 1 and 0.7% of the control cells, respectively), indicating that GW induced cell cycle arrest during cytokinesis.

Among the GW-treated *Giardia* trophozoites with two nuclei in the normal position, we found cells with condensed nuclei that which appeared larger and more deeply stained. Thus, the percentages of cells with condensed nuclei were also monitored in the cells treated with 5 µM GW for various lengths of time (3–24 h) (Fig. 1b). The percentage of cells with condensed nuclei significantly increased in cells treated with 5 μM GW for 6 h compared to DMSO-treated cells.

In order to determine the effect of GlPLK inhibition on DNA content of *Giardia*, we treated trophozoites with 0.1% DMSO or 5 μM GW for various lengths of time (6, 12, 18 and 24 h) and then analyzed the tropozoites by flow cytometry (Fig. 1c). The presented data are representatives of three independent samples per each condition. Control cells (untreated cells and DMSO-treated cells) were found to be a mixture of G1/S-, and G2/M-phase cells, with the cells at the G2/M phase the most dominant (72–73%), as reported previously [3]. For cells treated with GW for 6 and 12 h, the percentage of cells at G2/M phase increased to 86%, which was not a statistically significant difference. Interestingly, a greater number of cells treated with 5 μM GW for longer times, 18 and 24 h, were present in the G1/S phase (63–66%), as compared with the control cells (26%). These results indicated that the inhibition of PLK in *Giardia* causes cell cycle arrest at the G2/M phase or the G1/S phase, depending upon the treatment time with GW.

**Fig. 2:**
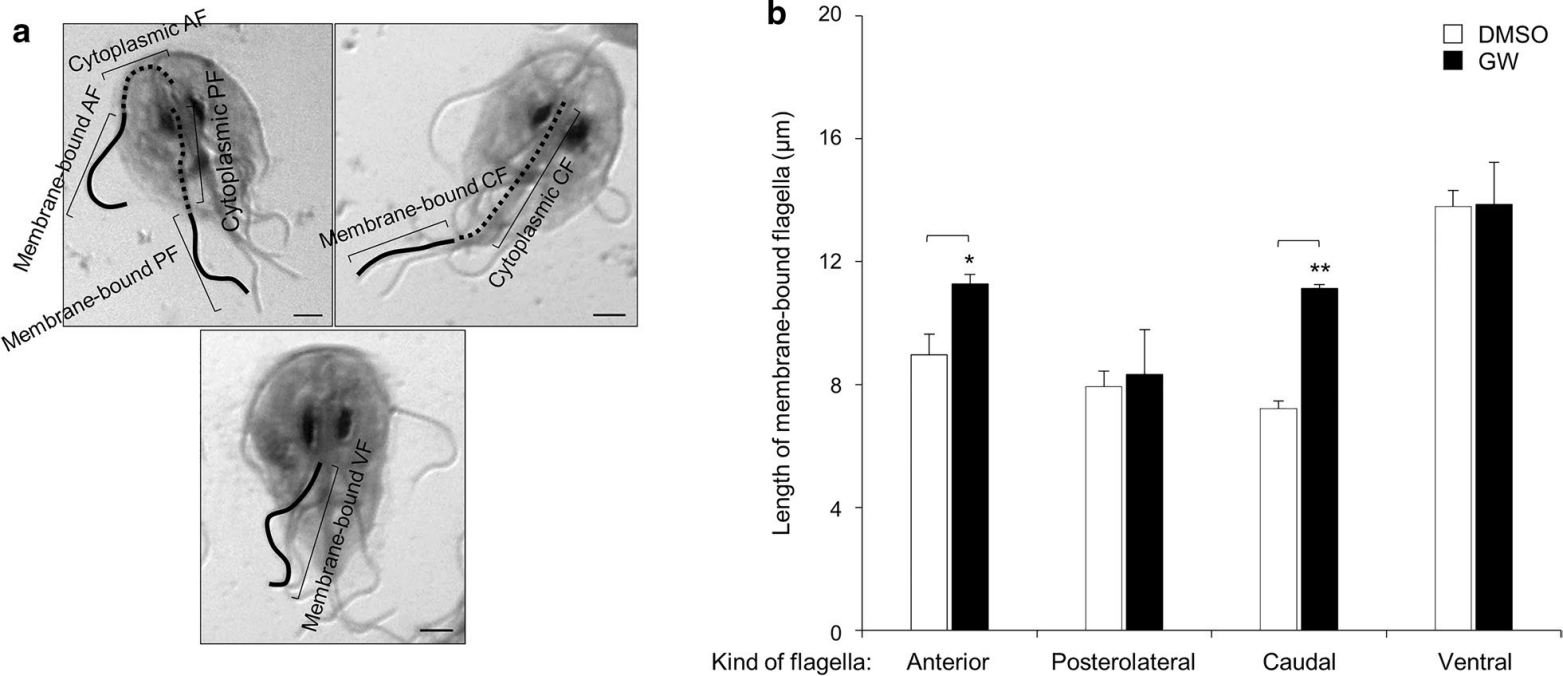
Effects of the PLK inhibitor GW on flagella formation in *G. lamblia*. Cells were treated with 5 µM GW (closed bars) or 0.1% DMSO (open bars) for 18 h, and then stained with Giemsa solution. **a** Representative figures showing how to measure the cytoplasmic anterior flagella (*AF*), membrane-bound AF, cytoplasmic posterolateral flagella (*PF*), membrane-bound PF, cytoplasmic caudal flagella (*CF*), membrane-bound CF and membrane-bound ventral F (*VF*). Scale bars: 2 μm. **b** Flagellar length was measured in 35 cells per each condition. Data are presented as the mean of three independent experiments. Asterisks indicate that difference is statistically significant at *P < 0.01 and **P = 0.01–0.05

In addition, we examined whether the GW treatment affected the flagella formation of *Giardia* trophozoites (35 cells per each condition, and 3 independent experiments) by quantitatively measuring the length of the membrane-bound and cytoplasmic portion of all four pairs of flagella (Fig. 2a). The cytoplasmic portion of the ventral flagella could not be measured in this assay. The length of the cytoplasmic posterolateral flagella was 5.2–7.2 μm. The anterior and caudal flagella had longer cytoplasmic portions (7.9–9.2 and 7.9–10.8 μm, respectively). None of the three flagella showed any significant change in the length of their cytoplasmic part in response to GW.

With respect to the membrane-bound region, the lengths of the four flagella were more variable. Under normal conditions, that is, DMSO treatment for various time periods, the membrane-bound ventral flagella were the longest (13.8 μm). The caudal flagella demonstrated the shortest membrane-bound region (7.2 μm). The lengths of the posterolateral and anterior membrane flagella were 7.9 and 9.0 μm, respectively. The effect of GW on the length of the membrane-bound flagella was determined using data derived from the cells treated with GW for 18 h and their counterpart control cells (Fig. 2b). GW treatment did not induce a significant increase in the length of the membrane-bound portion of the posterolateral and ventral flagella while, in contrast, GW-treated cells clearly showed an extension of the anterior and caudal flagella in their membrane-bound parts of up to 11.3 and 11.1 µm, respectively. These data clearly showed that the GW treatment affected the formation of these two flagella among the four types of flagella present in *G. lamblia*.

### Localization of GlPLK and definition of domains required for its localization in *Giardia* trophozoites

A homology search in the *Giardia* database indicated an ORF (GL50803_104150) as the putative *G. lamblia* PLK, GlPLK. Amino acid sequences deduced from the ORF were aligned with those of human and *Trypanosoma brucei* PLKs (GenBank accession numbers NP_005021.2 and Tb927.7.6310, respectively), showing 31–34% identity (Additional file 3: Fig. S2). The ORF was postulated to encode a protein of pI = 8.8, and a search of domains within this ORF using the Entrez program (http://www.expasy.org/) indicated that it contains a serine/threonine kinase domain (KD) at the amino-terminal portion (from amino acid residue no. 20 to 309). In addition, blocks of amino acids near the carboxyl terminus were proposed as the PBDs (amino acid residues no. 432–517 and 563–640), which had been conserved in diverse PLKs [23]. Based on the alignment of GlPLK with other PLKs, Lys51 was suggested as a residue that initially receives phosphate from ATP, and Thr179 and Thr183 residues were proposed as target sites that are subsequently phosphorylated.

**Fig. 3:**
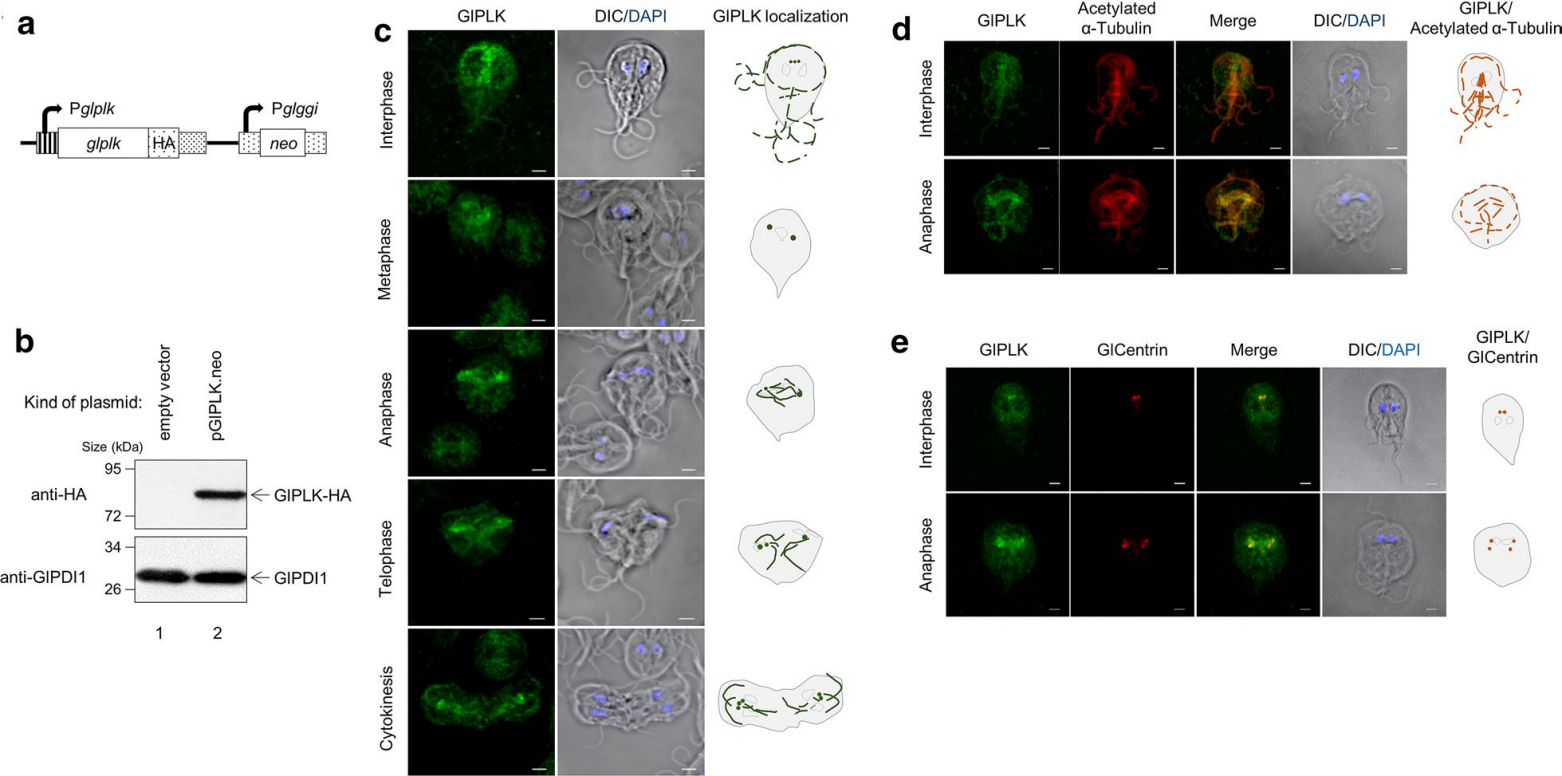
Expression and localization of *G. lamblia* PLK1 (GlPLK) in *G. lamblia*-expressing hemagglutinin (HA)-tagged GlPLK. **a** A schematic diagram of plasmid pGlPLK.neo. HA-tagged GlPLK was expressed from its own promoter, P*glplk*. Transfected trophozoites were selected by neomycin resistance conferred by the *neo* gene expressed by the P*glggi* promoter, a strong promoter of the γ-giardin gene. As a control, *Giardia* trophozoites were also transfected with pKS-3HA.neo, a vector control. **b** Western blotting to examine the expression of HA-tagged GlPLK. Extracts were prepared from *G. lamblia* containing empty vector (lane 1) or pGlPLK.neo (lane 2) and incubated with monoclonal mouse anti-HA antibodies. Membranes were first incubated in stripping buffer and then reacted with polyclonal rat antibodies specific to protein disulfide isomerase 1 (PDI1) of *G. lamblia*. **c** Localization of GlPLK. *Giardia lamblia* expressing HA-tagged GlPLK was probed with mouse anti-HA antibodies. The cells were then incubated with Alexa Fluor 488-conjugated anti-mouse IgG. Slides were mounted with ProLong™ Gold Antifade Mountant with the fluorescent stain DAPI, and then examined with a Zeiss LSM700 inverted confocal laser scanning microscope. Scale bars: 2 µm. **d** Co-localization of GlPLK and α-tubulin in *G. lamblia*. *Giardia* cells expressing HA-tagged GlPLK were probed with rat anti-HA antibodies and mouse anti-acetylated-α-tubulin monoclonal antibodies. **e** Co-localization of GlPLK and* G. lamblia* centrin (GlCentrin) in *G. lamblia*. Cells were reacted with mouse anti-HA antibodies and rat anti-GlCentrin polyclonal antibodies. Cells were then incubated with Alexa Fluor 555-conjugated anti-rat IgG and Alexa Fluor 488-conjugated anti-mouse IgG. A differential interference contrast image was acquired to show cell morphology. Scale bars: 2 μm.* DIC* Differential interference contrast

A plasmid, pGlPLK.neo, was constructed (Fig. 3a) and used to construct transgenic *Giardia* trophozoites expressing HA-tagged GlPLK. Western blotting of the resulting *G. lamblia* extracts confirmed the expression of HA-tagged GlPLK as an immunoreactive band with a molecular weight of 80 kDa (Fig. 3b). In contrast, the extracts of *G. lamblia* carrying the vector control, pKS-3HA.neo, did not produce any immunoreactive bands in the same analysis. Western blotting of the same membrane with anti‐GlPDI1 antibodies [24] served as a loading control for the total amount of protein in the extracts used for this assay.

The localization of GlPLK was determined using *Giardia* expressing HA‐tagged GlPLK (Fig. 3c). GlPLK was found in the basal bodies, flagella, axonemes, an adhesive disc and median bodies of *Giardia* trophozoites at interphase. Localization at the basal bodies was maintained in the dividing cells (cells at metaphase, anaphase and telophase), as well as at cytokinesis. In cells at anaphase, GlPLK was also present in the mitotic spindles and axonemes of the dividing cells.

To confirm the localization of GlPLK, *Giardia* cells expressing HA-tagged GlPLK were double‐stained for GlPLK and microtubules (MTs) using anti‐HA and anti‐acetylated-α‐tubulin antibodies, respectively (Fig. 3d). In *Giardia* cells at interphase and anaphase, GlPLK was found together with MTs in the basal bodies, axonemes, median bodies and flagella. *Giardia* cells at anaphase also demonstrated the co-localization of GlPLK with MTs in the mitotic spindles present between two separated groups of basal bodies.

Basal bodies serve as the MT-organizing center (MTOC) in *G. lamblia* [25], and the MTOC can be observed by staining for its marker, centrin. Additional IFAs for *Giardia* expressing HA-tagged GlPLK were performed using antibodies against HA and *G. lamblia* centrin (GlCentrin) (Fig. 3e). These double‐stained *Giardia* cells clearly showed the co‐localization of GlPLK and GlCentrin during cell division as well as at interphase.

**Fig. 4:**
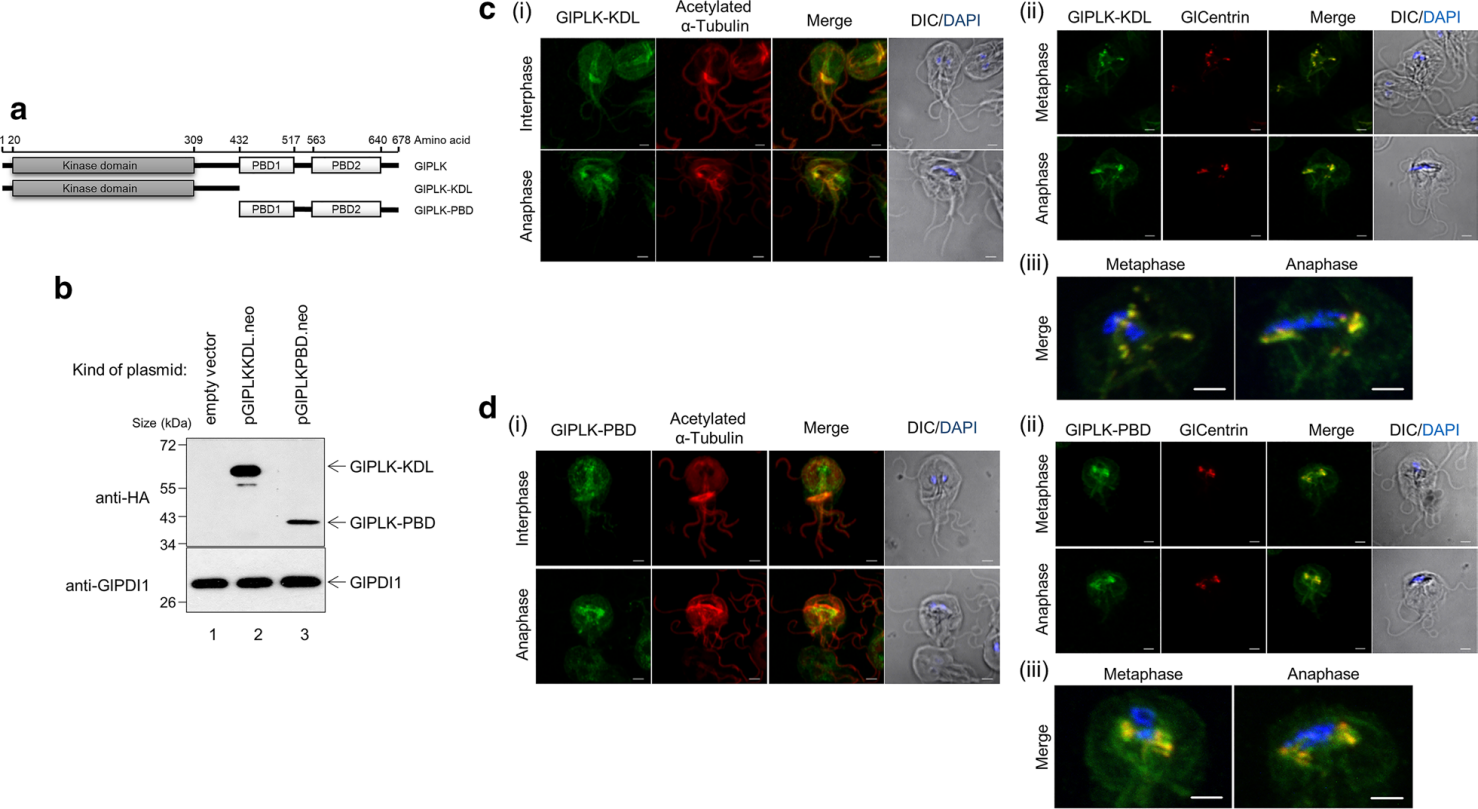
Expression and localization of truncated GlPLKs in *G. lamblia*. **a** A schematic diagram of plasmids pGlPLKKDL.neo and pGlPLKPBD.neo. Two truncated GlPLK proteins are expressed in an HA-tagged form from their own promoter, P*glplk*. Plasmid pGlPLKKDL encodes GlPLK with the KD and linker region, whereas pGlPLKPBD contains DNA coding for the polo-box domains (PBDs) of GlPLK. Plasmid pKS-3HA.neo was transfected into *Giardia* trophozoites as a control. **b** Western blotting to examine the expression of HA-tagged truncated GlPLKs. Extracts were prepared from *G. lamblia* containing empty vector (lane 1), pGlPLKKDL.neo (lane 2), or pGlPLKPBD.neo (lane 3), and incubated with monoclonal mouse anti-HA antibodies. Membranes were incubated in stripping buffer, and then reacted with polyclonal rat antibodies specific to GlPDI1. **c** Co-localization of GlPLK-KDL with α-tubulin (**i**) or GlCentrin (**ii**, **iii**). *Giardia lamblia* cells expressing HA-tagged truncated GlPLK-KDL were probed with rat anti-HA antibodies and mouse anti-acetylated-α-tubulin monoclonal antibodies. Otherwise, these cells were reacted with rat anti-GlCentrin polyclonal antibodies instead of anti-acetylated-α-tubulin monoclonal antibodies. Panel **iii** is an extended view of panel **ii**. Incorrectly positioned basal bodies are indicated with white arrows. **d** Co-localization of GlPLK-PBD with α-tubulin (**i**) or GlCentrin (**ii**, **iii**). *G. lamblia* cells expressing HA-tagged truncated GlPLK-PBD were probed with rat or mouse anti-HA antibodies along either with mouse anti-acetylated-α-tubulin antibodies (**i**), or rat anti-GlCentrin polyclonal antibodies (**ii**,** iii**), respectively. Panel **iii** is an extended view of panel **ii**. The cells were then incubated with Alexa Fluor 488-conjugated anti-rat IgG and Alexa Fluor 568-conjugated anti-mouse IgG (α-tubulin co-localization) or Alexa Fluor 555-conjugated anti-rat IgG and Alexa Fluor 488-conjugated anti-mouse IgG (for centrin co-localization). Slides were mounted with ProLong™ Gold Antifade Mountant with DAPI, and then examined with a Zeiss LSM700 inverted confocal laser scanning microscope. A differential interference contrast image was acquired to show cell morphology. Scale bars: 2 μm

As mentioned above, GlPLK comprises two regions: the KD and two PBDs (Fig. 4a). The region between the KD and PBDs was named the linker. To examine whether the KD and/or PBDs play a role in GlPLK localization, we constructed two plasmids, pGlPLKKDL.neo and pGlPLKPBD.neo, that expressed the KD linker and the PBDs of GlPLK, respectively. Western blotting using anti-HA antibodies revealed the expression of the truncated GlPLK proteins, KD linker and PBDs, in *Giardia* trophozoites in the form of immunoreactive bands with a molecular weight of 60 and 40 kDa, respectively (Fig. 4b). On the other hand, *Giardia* carrying the vector plasmid pKS-3HA.neo did not show any immunoreactive bands.

*Giardia lamblia* cells carrying pGlPLKKDL.neo were double-stained with anti-HA and anti-acetylated-α-tubulin antibodies or with anti-HA and anti-GlCentrin antibodies (Fig. 4c) in order to observe whether this truncated GlPLK-KDL was correctly localized in mitotic spindles and basal bodies, respectively. In interphase *Giardia* cells, double staining with antibodies against acetylated-α-tubulin and HA resulted in the labeling of flagella, axonemes, a median body and basal bodies (*n* = 5). In contrast, GlPLK-KDL was not found in the mitotic spindles of the dividing *Giardia* at anaphase (*n* = 5). Double staining of *G. lamblia* cells carrying pGlPLKKDL.neo with anti-HA and anti-GlCentrin antibodies revealed the co-localization of these two proteins in basal bodies [cells at metaphase (*n* = 2) and cells at anaphase (*n* = 6)]. However, the relative position of these structures with DAPI-stained nuclei indicated an incorrect localization of the basal bodies, as presented in an extended view.

In contrast, *Giardia* cells expressing truncated GlPLK-PBD demonstrated the same pattern of co-localization with the full-length GlPLK with respect to α-tubulin (Fig. 4d). In addition to being found in the flagella, axonemes and median bodies of the interphase cells (*n* = 3), GlPLK-PBD was found at the mitotic spindles in dividing *Giardia* cells (*n* = 7). Double staining of *Giardia* cells expressing GlPLK-PBD revealed the co-localization of this protein with GlCentrin in basal bodies (*n* = 7). Extended views of these dividing cells indicated that double-stained basal bodies positioned themselves in the correct positions for mitosis. These results suggest that the PBD of GlPLK is required for GlPLK localization in mitotic spindles and the correct positioning of basal bodies during *Giardia* cell division.

### Effect of GlPLK knockdown on cell division and flagella biogenesis in *G. lamblia*

**Fig. 5:**
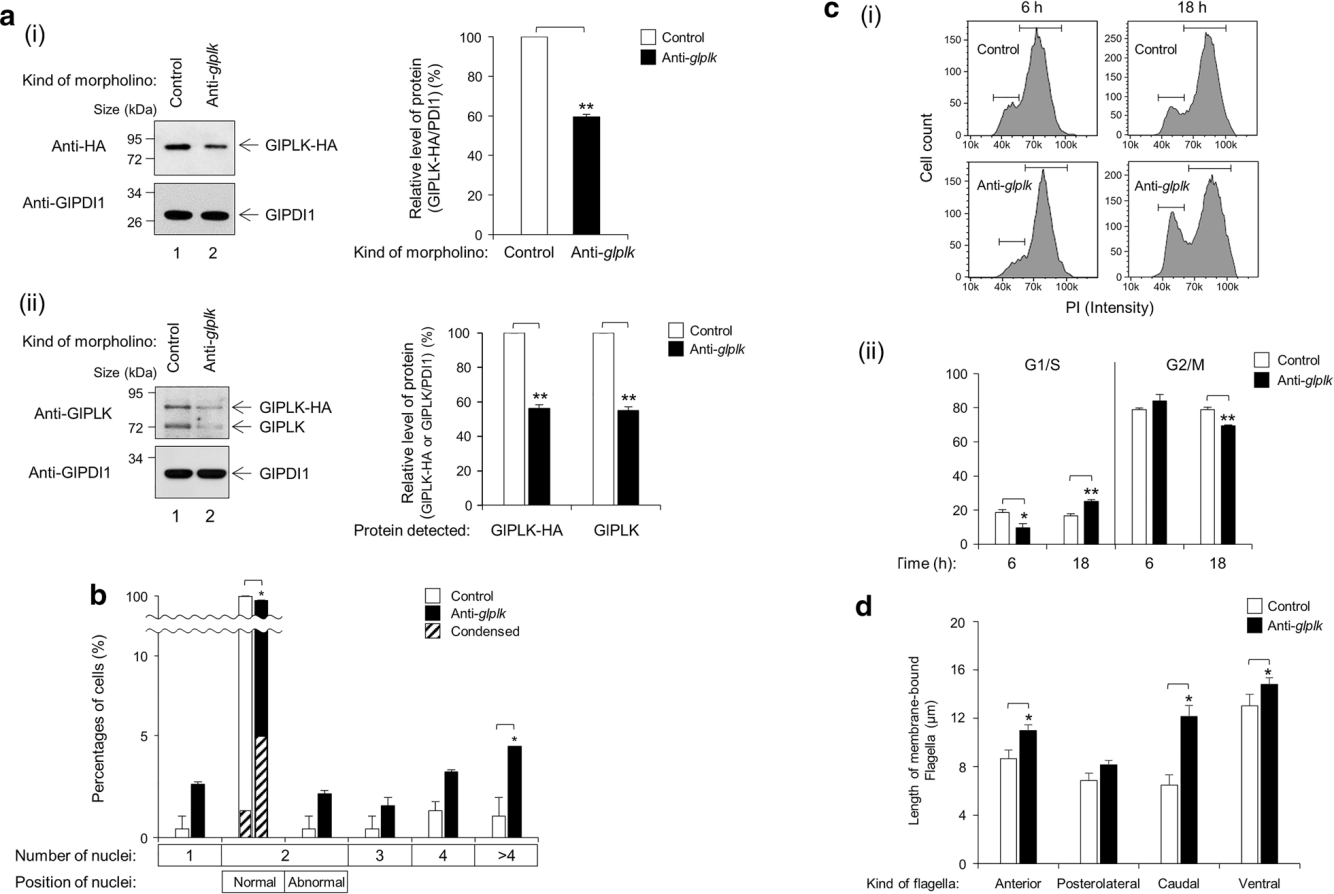
Effect of morpholino-mediated GlPLK knockdown in cell division and flagella formation in *G. lamblia*. *Giardia* trophozoites expressing HA-tagged GlPLK were collected at 18 h after electroporation with control (open bars) or anti-*glplk* morpholino (closed bars). **a** Morpholino-mediated GlPLK knockdown in *G. lamblia*. (**i**) Western blot analysis using anti-HA or anti-GlPDI1 antibodies, (**ii**) western blot analysis using anti-GlPLK or anti-GlPDI1 antibodies. The relative expression of HA-tagged GlPLK (**i**) or/and endogenous GlPLK (**ii**) in extracts of cells treated with an anti-*glplk* morpholino compared with the expression in the control cells is presented as a bar graph. **b** Effects of morpholino-mediated GlPLK knockdown on the nuclear phenotypes of *G. lamblia*. The cells transfected with control (open bars) or an anti-*glplk* morpholino (closed bars) were maintained for 18 h prior to staining with Giemsa solution. At least 300 cells were examined for the number and position of nuclei under each condition using an Axiovert 200 microscope. Among the cells with two nuclei in the normal position, the number of cells showing nuclear condensation was also recorded (hatched bars). **c** Effects of morpholino-mediated GlPLK knockdown on the ploidy of their DNA of *G. lamblia*. The cells transfected with control (open bars) or an anti-*glplk* morpholino (closed bars) were maintained for 18 h. After being stained with PI, the ploidy of their DNA was analyzed by flow cytometry. **d** Effects of morpholino-mediated GlPLK knockdown on flagella formation in *G. lamblia*. Flagella length was measured in 35 cells per condition. Data are presented as an average of three independent experiments. Asterisks indicate that the difference is statistically signiicant at *P = 0.01–0.05 and **P < 0.01, respectively

To define the role of this putative GlPLK in *G. lamblia*, we designed an anti-*glplk* morpholino to block the translation of *glplk* mRNAs (Table 1). A control morpholino (non-specific oligomer) was also synthesized and transfected by electroporation into *G. lamblia* trophozoites carrying pGlPLK.neo (Table 2). When the cells were harvested at various time-points, ranging from 12 to 48 h, and analyzed for GlPLK inhibition, the cells at 24 h post-transfection demonstrated a maximal inhibition of GlPLK expression (data not shown). However, we chose the cells harvested at 18 h after transfection for further studies (Fig. 5a). In cells treated with an anti-*glplk* morpholino, the amount of HA-tagged GlPLK at 18 h post-transfection had decreased to 59.5% of that in cells treated with the control morpholino (*P* = 0.0003). In addition, these extracts were examined to determine their intracellular GlPLK-HA and GlPLK levels by western blotting using anti-GlPLK antibodies. In cells treated with an anti-*glplk* morpholino, the amounts of GlPLK-HA and GlPLK at 18 h post-transfection had decreased to 56 and 55% of those in cells treated with the control morpholino, respectively.

The effect of GlPLK knockdown on cell division was determined based on the nuclear phenotypes, which included the number of nuclei and the condensation of the DNA in the cells (Fig. 5b). The percentage of cells with two normally positioned nuclei decreased from 98.7 to 90.7%. Among these cells, the number of cells showing nuclear condensation increased from 0.4 to 4.6%. The percentages of cells with one nucleus, two nuclei in abnormal position, three nuclei and four nuclei were slightly increased in cells treated with an anti-*glplk* morpholino, without any statistical significance. Only the percentage of cells with more than four nuclei showed a statistically significant increase in anti-*glplk* morpholino-treated cells (*P* = 0.02).

The effect of GlPLK depletion on the DNA ploidy of *Giardia* cells was also determined by flow cytometric analysis (Fig. 5c). Cells treated with control morpholino for 6 or 18 h showed a similar proportion of cells in the G1/S and G2/M phases (19 and 79%, respectively). In cells treated with anti-*glplk* morpholino, the percentage of G2/M-phase cells increased to 84% after 6 h post-transfection. In contrast, a greater number of cells treated with anti-*glplk* morpholino for 18 h were present in the G1/S phase (25%), as compared with the control cells (17%). The percentage of G2/M-phase cells decreased to 69% after 18 h post-transfection of anti-*glplk* morpholino, compared 79% in control cells.

GlPLK depletion also resulted in the formation of *Giardia* trophozoites with longer flagella (Fig. 5d). The length of the membrane-bound portion of the caudal flagella in cells treated with an anti-*glplk* morpholino increased to 12.1 μm compared to 6.5 μm in the control morpholino cells (*P* = 0.003). GlPLK-depleted cells also showed extension of the anterior and ventral flagella in their membrane-bound parts of up to 11.0 and 14.8 µm compared to 8.7 and 13.0 µm, respectively, in control cells.

### Expression pattern of GlPLK at G1/S and G2/M phase of the *Giardia* cell cycle

As human PLK1 is highly expressed during mitosis [26], we examined whether GlPLK expression varies in a phase-dependent manner. *Giardia* cells were treated with nocodazole to prepare G2/M-phase cells (91%) or sequentially with nocodazole and aphidicolin to acquire G1/S-arrested cells (86%). The stage of the resulting *Giardia* cells carrying pGlPLK.neo was confirmed by flow cytometry (Additional file 4: Fig. S3A). Control *Giardia* trophozoites treated with 0.01% DMSO were found to be a mixture of G1/S- and G2/M-phase cells, G2/M-phase cells being the dominant cell type (78%).

Western blotting of these cell extracts using anti-HA antibodies demonstrated an increased amount (1.5-fold) of GlPLK in G2/M-phase and interphase cells in comparison with G1/S-phase cells (Additional file 4: Fig. S3B). The immunoreactive band was absent from the extracts prepared from *Giardia* cells carrying pKS-3HA.neo. Western blotting of the same blot using anti-GlPDI1 antibodies served as a loading control.

Increased expression of the *glplk* transcript was also examined using an alternative method, quantitative reverse transcription (RT)-PCR (Additional file 4: Fig. S3C). The relative level of *glplk* transcripts to *glactin* transcripts remained increased (2.4-fold) in G2/M-phase cells compared to G1/S-phase cells (*p* = 0.01). To monitor the phases of our samples, the assays included two G1/S phase marker genes encoding histone H3 and histone H4, which showed a decreased expression in the G2/M-phase cells [5]. In addition, the transcript level of Glγ-giardin was measured in these cells and found to show increased expression in G2/M phase, as expected [16].

### Subcellular localization of GlPLK in *G. lamblia*

In order to function properly during mitosis, PLK1 should be localized to specific sites through differential interaction with various scaffold proteins [23]. The nucleus is one of the subcellular locations where PLK1 localizes in the G2 phase [27].

*Giardia* extracts were prepared from *Giardia* cells expressing HA-tagged GlPLK at interphase, the G1/S phase and the G2/M phase, and then further divided into cytoplasmic and membrane fractions, which may include nuclear fractions. These extracts were analyzed by western blotting using anti-HA antibodies (Additional file 5: Fig. S4). In addition, extracts were evaluated for *G. lamblia* glyceraldehyde 3-phosphate dehydrogenase (Gl50803_6687; GlGAP1), *G. lamblia* centrin (Gl50803_104685; GlCentrin) and *G. lamblia* centromeric histone H3 (GL50803_20037; GlCenH3) as markers for cytoplasmic, membrane and nuclear proteins, respectively. Because the amino acid sequence alignment of the three histone H3 proteins of *G. lamblia* demonstrated 33–46% identity among them, it is unlikely that anti-GlCenH3 reacts with the other two histone H3 proteins. Even though there is some cross-reactivity of these antibodies against the other histone H3 proteins, it did not interfere with this experiment in that all three hisone H3 proteins are located in the nuclei of *G. lamblia* [20].

GlPLK was found in both the cytoplasmic and membrane fractions in all examined phases. As expected, GlGAP1 was mainly present in the cytoplasmic fraction, and GlCentrin and GlCenH3 were found only in the membrane fraction.

Both G1/S- and G2/M-phase cells demonstrated GlPLK localization in the membrane fraction, and more GlPLK was found in the G2/M-phase cells than in the G1/S-phase cells. A constant amount of GlGAP1 was present in the cytoplasmic fraction of all examined phases, whereas more GlCentrin and GlCenH3 were found in the membrane fraction of the G2/M-phase cells than in the G1/S-phase cells.

### Expression and localization of phosphorylated GlPLK in *G. lamblia*

**Fig. 6:**
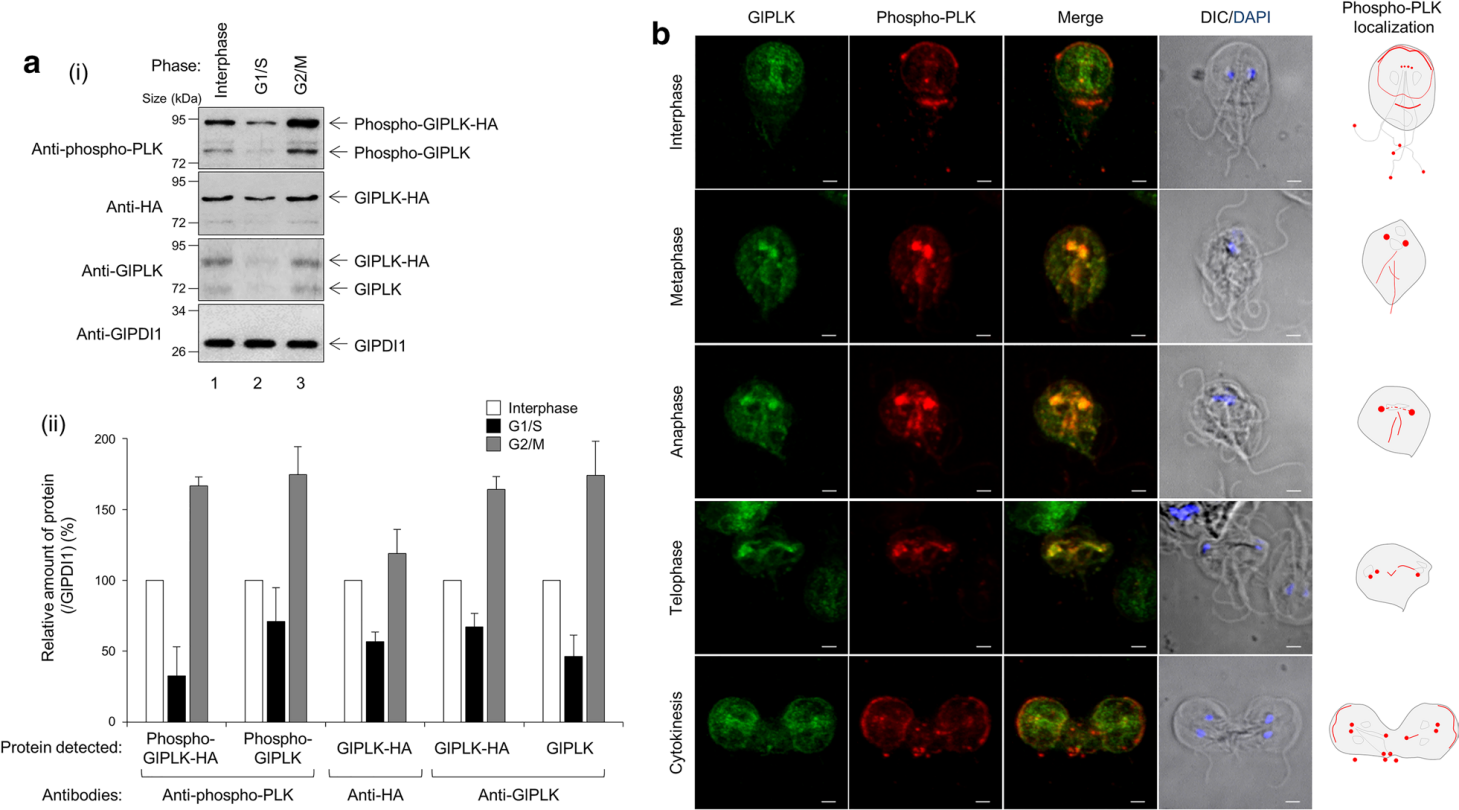
Expression and localization of phosphorylated GlPLK in *G. lamblia*.** a** *Giardia lamblia* cells expressing HA-tagged GlPLK in the interphase (lane 1), G1/S phase (lane 2), and G2/M phase (lane 3). Western blot analysis. Extracts of these cells were probed with anti-phospho-PLK, anti-HA, anti-GlPLK or anti-GlPDI1 antibodies. Levels of phospho-GlPLK-HA, phospho-GlPLK, GlPLK-HA and GlPLK were normalized to GlPDI1, a protein loading control. Amounts of these GlPLK proteins are expressed as relative values to those observed in the interphase cells. The presented western blot is a representative of three independent experiments, and averages of three experiments are presented as a bar graph. **b** Localization of GlPLK and phosphorylated GlPLK in *Giardia*. *G. lamblia* expressing HA-tagged GlPLK were incubated with antibodies specific to the phosphorylated form of PLK (1:100) along with anti-HA antibodies. These cells were then reacted with anti-Alexa Fluor 568-conjugated anti-mouse IgG and Alexa Fluor 488-conjugated anti-rat IgG. Slides were mounted with ProLong™ Gold Antifade Mountant with DAPI, and then examined with a Zeiss LSM700 inverted confocal laser scanning microscope. Scale bars: 2 µm

In addition to the expression of GlPLK, GlPLK activity is important for its role in *Giardia* cell division; this role may be regulated by its activation status, possibly by phosphorylation. We examined whether GlPLK phosphorylation was modulated in a cell phase-dependent manner (Fig. 6a). *Giardia* cells carrying pGlPLK.neo were used to prepare the cell extracts at interphase, G1/S phase and G2/M phase, and then analyzed by western blotting using anti-phospho-PLK, anti-HA, anti-GlPLK and anti-GlPDI1 antibodies. In the western blot analysis with anti-phospho-PLK, both HA-tagged GlPLK and endogenous GlPLK were detected, and the amount of both proteins increased fivefold and 2.5-fold during the G2/M phase, respectively. In the same manner, western blotting using anti-GlPLK and anti-HA antibodies demonstrated more than a twofold increase in the expression of both HA-tagged GlPLK and endogenous GlPLK during the G2/M phase.

*Giardia* trophozoites were double-stained with anti-HA and anti-phospho-PLK antibodies (Fig. 6b). Both anti-HA and anti-phospho-PLK antibodies stained basal bodies in the interphase and dividing cells. Localization of phospho-GlPLK in the cytoplasmic portion of anterior flagella, median bodies, and flagella tips was distinct. In dividing cells, phospho-GlPLK was also found at mitotic spindles, as with the HA-tagged GlPLK.

### *In vitro* autophosphorylation of GlPLK and identification of critical amino acid residues for its autophosphorylation

**Fig. 7:**
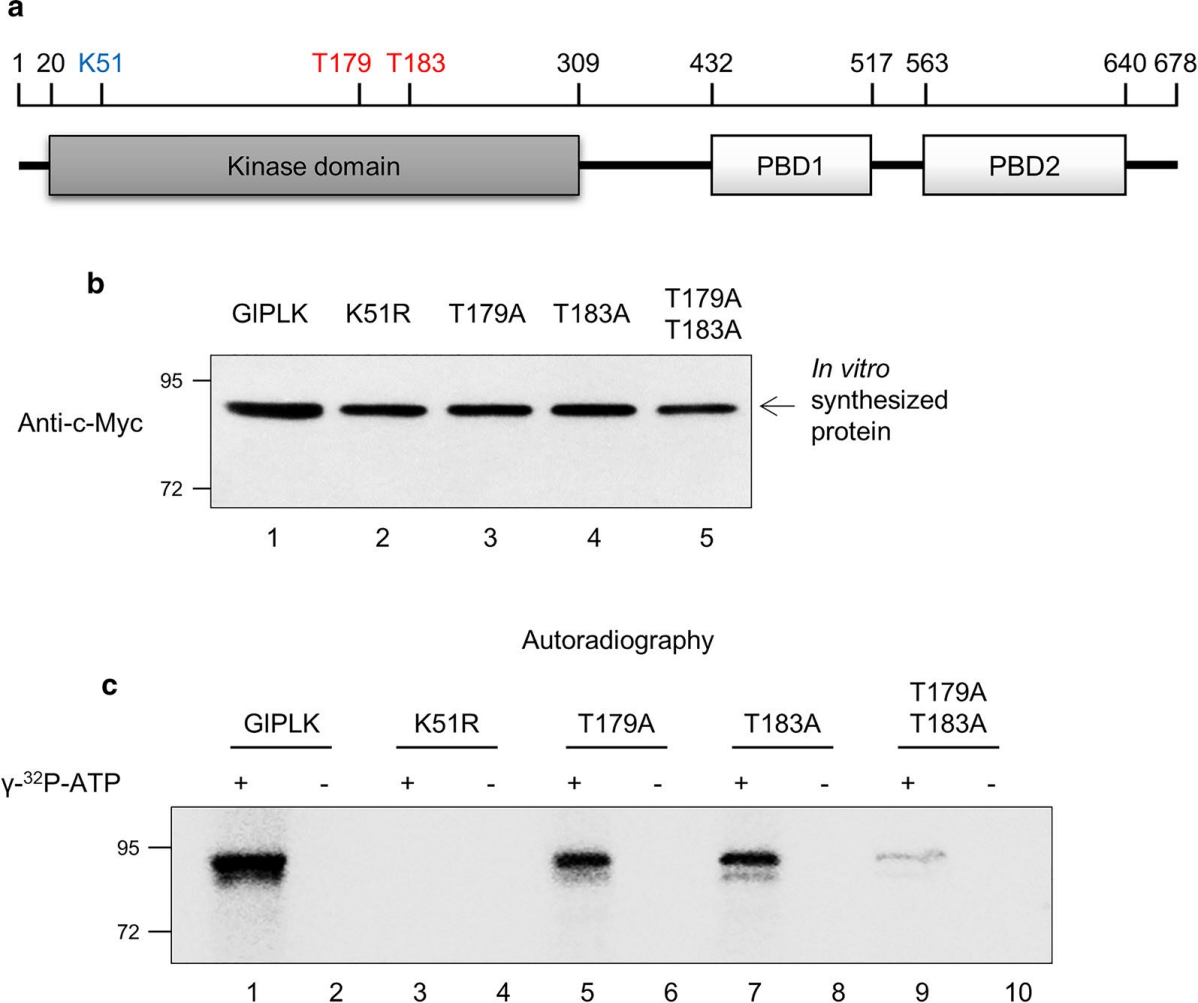
*In vitro *autophosphorylation of wild-type and mutant GlPLKs. **a** A schematic diagram of GlPLK. Serine/threonine kinase (KD) and two polo-box domains (*PBD1*,* PBD2*) are indicated as boxes. Lys51 (*K51*) is suggested as a residue that initially receives phosphate from ATP, and two threonine residues (*T179* and* T183*) are proposed as the target sites of subsequent phosphorylation. **b** Western blot of rGlPLK proteins synthesized* in vitro* using mouse anti-c-Myc antibodies (1:1000). **c** Phosphorylation of* in vitro*-synthesized GlPLKs. The c-Myc-tagged GlPLKs (wild-type GlPLK, K51R mutant GlPLK, and T179AT183A mutant GlPLK) were prepared* in vitro* and then used for kinase assays. GlPLK proteins were resuspended in 20 μl kinase buffer in the presence of 2.5 µCi [γ-^32^P]ATP. The kinase reactions were then subjected to 12% SDS-PAGE and visualized by autoradiography

The putative amino acid sequence of GlPLK indicates a serine/threonine KD at the amino terminus and two PBDs at the carboxyl terminus (Fig. 7a). Based on comparison with other PLKs, it was predicted that Lys51 is the primary binding site for ATP, and that the phosphate of Lys51 is eventually transferred to Thr179 and Thr183 in the activation loop.

Kinase assays were also performed using recombinant GlPLK (rGlPLK), which was synthesized using* in vitro* transcription and translation systems, and expression was confirmed by western blotting with anti-c-Myc antibodies (Fig. 7b). Upon incubation with [γ-^32^P]ATP, rGlPLK was radiolabeled due to autophosphorylation.

To define the amino acid residues that are critical for GlPLK autophosphorylation, several recombinant GlPLK proteins were synthesized using* in vitro* transcription/translation systems and used for kinase assays (Fig. 7c). Specifically, the two putative phosphorylation sites were mutated to Ala, and the resulting mutant GlPLK proteins (GlPLKT179A and GlPLKT183A) were used for kinase assays. In an additional mutant GlPLK, the putative ATP binding site of Lys51 was mutated to Arg (GlPLKK51R). Both GlPLKT179A and GlPLKT183A proteins were autophosphorylated, although the efficiency of autophosphorylation was lower than that of wild-type GlPLK. When both Thr179 and Thr183 were mutated to Ala in GlPLK, the resulting protein exhibited a dramatic decrease in its autophosphorylation ability. Conversion of Lys51 to Arg abolished the autophosphorylation of rGlPLK. This result demonstrated that both Thr179 and Thr183 in the activation loop of GlPLK were phosphorylated. As expected, Lys51 of GlPLK was confirmed to serve as an ATP binding site.

### Role of GlPLK phosphorylation in cytokinesis and flagella biogenesis in *G. lamblia*

**Fig. 8:**
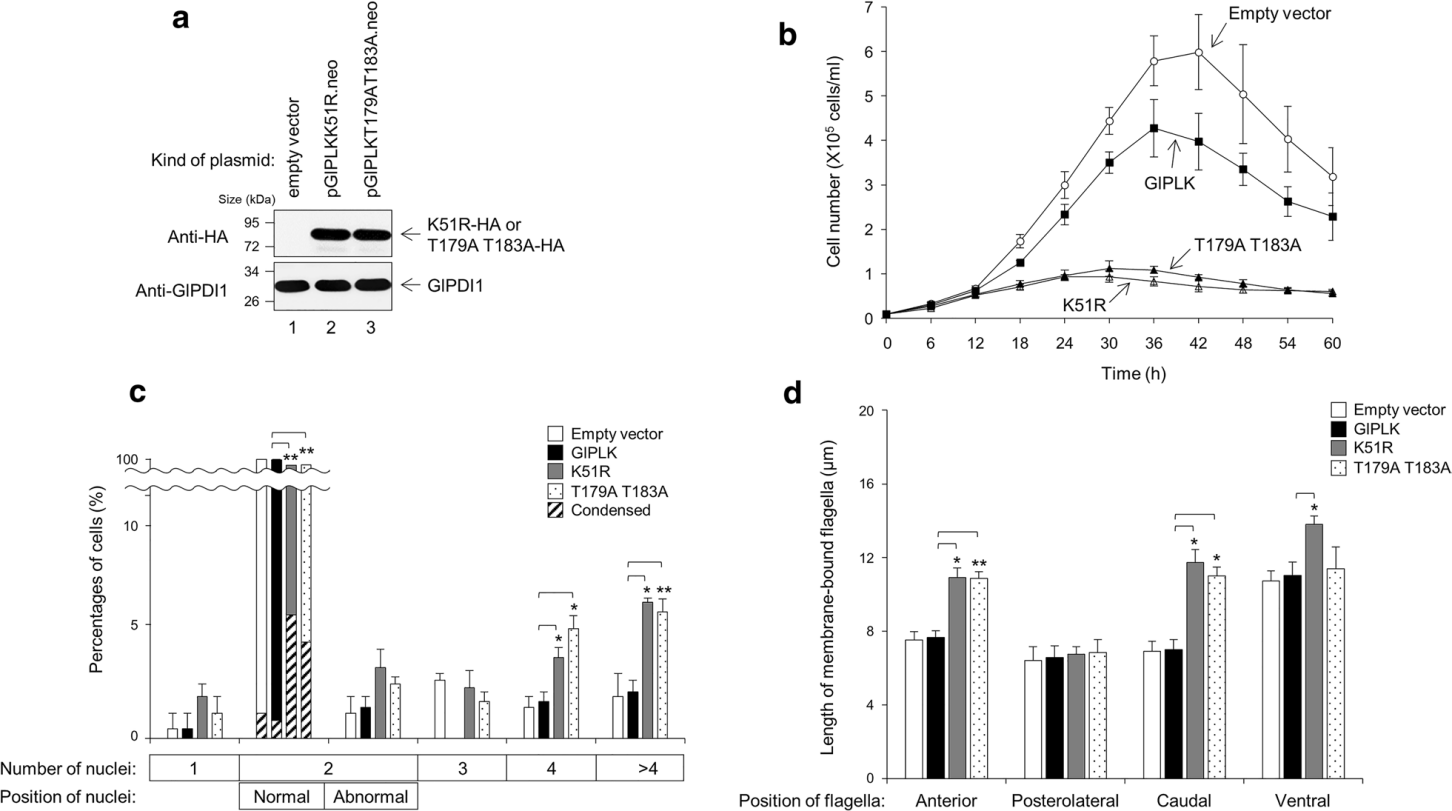
Functional defects caused by ectopic expression of mutant GlPLKs in *Giardia*. *Giardia* carrying the empty vector (pKS-3HA.neo), and *Giardia* expressing wild-type GlPLK, K51R mutant GlPLK, or T178AT183A mutant GlPLK, were constructed by electroporation of the corresponding plasmid into *Giardia* trophozoites. **a** Western blot analysis showing the expression of HA-tagged mutant GlPLK proteins in *Giardia*. Cell extracts were prepared from *Giardia* carrying the vector plasmid (lane 1), *Giardia* expressing K51R mutant GlPLK (lane 2), or T178AT183A mutant GlPLK (lane 3), and reacted with anti-HA antibodies. **b** Growth curves of *Giardia* carrying the vector plasmid (open circles) or expressing wild-type GlPLK (closed squares), K51R mutant GlPLK (open triangles) or T178AT183A mutant GlPLK (closed triangles). The number of parasites per milliliter was determined using a hemocytometer. Each experiment comprised three cultures and was repeated three times using independently obtained transfected cells. **c** Effects of the ectopic expression of mutant GlPLKs on the nuclear phenotypes of *G. lamblia*. Various cells, *Giardia* carrying the vector plasmid (open bars) and *Giardia* expressing wild-type GlPLK (closed bars), K51R mutant GlPLK (gray bars) or T178AT183A mutant GlPLK (dotted bars), were stained with Giemsa solution. At least 300 cells were examined for the number and position of the nuclei under each condition using an Axiovert 200 microscope. Among the cells with two nuclei in the normal position, the number of cells showing nuclear condensation was also recorded (hatched bars). **d** Effects of the ectopic expression of mutant GlPLKs on flagella formation in *G. lamblia*. Flagella length was measured in 35 cells per condition. Data are presented as an average of three independent experiments. Asterisks indicate that the difference is statistically signiicant at *P = 0.01–0.05 and **P < 0.01, respectively

Subsequent experiments were performed to determine the physiological roles of GlPLK. Transgenic *G. lamblia* carrying pGlPLKK51R.neo was constructed. In addition, *Giardia* cells ectopically expressing mutant GlPLK (T179AT183A) were prepared. Western blotting demonstrated that the transgenic cells expressed HA-tagged GlPLK proteins (Fig. 8a).

The growth of various *Giardia* cells (ectopically expressing GlPLK, mutant GLPLKK51R, mutant GlPLKT178AT183A or carrying empty vector) was determined (Fig. 8b). The growth of *Giardia* cells overexpressing wild-type GlPLK was slightly affected when compared with that of the control cells. Interestingly, *Giardia* cells expressing mutant GlPLKs showed dramatic growth inhibition compared to those carrying the vector plasmid.

These cells were then evaluated for their number of nuclei as described earlier (Fig. 8c). The majority of the control cells carrying vector plasmid and cells overexpressing wild-type GlPLK (98%) had two nuclei in the correct position. The percentage of cells with two nuclei in the correct position were decreased to 88–89% when mutant GlPLKs were ectopically expressed. Among these cells, the number of cells showing nuclear condensation increased to 4.7 and 2.6% from 0.2 to 0.3% (control and GlPLK-overexpressing cells, respectively) in the case of *Giardia* expressing GLPLKK51R and GlPLKT179AT183A. In contrast to a slight increase in the number of cells with one nucleus, two abnormally located nuclei and three nuclei, the cells expressing mutant GlPLKs showed a dramatic increase in percentage of cells carrying four nuclei and more than four nuclei. These results indicate that Lys51, as well as two Thr residues (Thr179 and Thr183), in GlPLK may play a role in cell division in *Giardia*. GlPLK overexpression did not affect the length of flagella. In contrast, the ectopic expression of mutant GlPLK resulted in the extension of the lengths of three flagella, except for the posterolateral flagella (Fig. 8d). These data indicate that GlPLK plays a role in regulating flagella morphogenesis and cell cycle in *Giardia* and that GlPLK phosphorylation is critical for its* in vivo* function.

## Discussion

Mammalian PLK is a multi-faceted kinase that controls several steps of the cell cycle [28]. In contrast to the presence of PLK paralogues in other systems, *G. lamblia* seems to have one PLK, the function of which was demonstrated in the present study with a chemical inhibitor of PLK (Additional file 2: Table S1; Figs. 1, 2) and an anti-*glplk* morpholino (Fig. 5).

Since the phenotype we monitored was the number of nuclei of GlPLK-inhibited and GlPLK-depleted *Giardia* trophozoites (Additional file 2: Table S1; Figs. 1a, 5b), these experiments only provide evidence for the role of GlPLK in cytokinesis, but not in other aspects of the cell cycle, such as centrosome maturation, kinetochore formation and mitotic spindle function. However, the increased percentage of cells with condensed DNA among the cells with two nuclei under these conditions (Figs. 1b, 5a) suggested that GlPLK plays an important role in mitosis similar to that of mammalian PLK1 [23]. This suggestion was further strengthened by IFAs showing the localization of GlPLK and phospho-GlPLK at basal bodies and mitotic spindles in dividing cells (Figs. 3c–e, 6b, respectively). GlPLK localization at basal bodies and mitotic spindles was confirmed by co-localization experiments using marker proteins (Fig. 3d, e).

The meaning of the results related to the effect of GlPLK inhibition on DNA ploidy of *Giardia* trophozoites (Fig. 1c) is difficult to interpret in that the arrested phases varied depending upon the treatment time with GW. At 6 and 12 h post- GW treatment, more *Giardia* trophozoites were found at the G2/M phase, whereas majority of cells were arrested at G1/S phase after 18 h and 24 h post-GW treatment. Therefore, it is premature to draw any conclusion on the role of GlPLK in a specific phase of the *Giardia* cell cycle. Considering the generation time for *Giardia* trophozoites, i.e. 4 h, the results showing an increased number of cells at the G2/M phase at 6 and 12 h are more plausible. However, we cannot rule out the possibility that GlPLK also plays a role in the G1/S phase. Flow cytometric analysis on GlPLK-depleted *Giardia* using anti-*glplk* morpholino also demonstrated a similar pattern of time-dependent arrests even though degrees of arrests are less than those with GW treatment (Fig. 5c). In contrast to the restricted functions of PLK2, PLK3 and PLK5 in non-proliferating vertebrate cells [29], PLK1 and PLK4 are highly conserved. PLK1 is a multi-functional kinase involved in mitosis and cytokinesis, whereas PLK4 is known to function as a centriole assembly factor in the S phase [30]. It is possible that GlPLK functions as a combined form of PLK1/PlK4.

Since the subcellular localization of PLK occurs* via* interactions with various scaffold proteins and is important for its functions in other systems [31], *Giardia* extracts were fractionated into cytoplasmic and membrane fractions in order to examine whether GlPLK is localized in a certain fraction (Additional file 5: Fig. S4). GlPLK was found in both fractions under any of the conditions. Most notably, IFAs showed no evidence of the nuclear localization of GlPLK (Figs. 3c, 6b). However, the subcellular fractionation assay showed that GlPLK was present in the membrane fractions, which contained the nuclear protein marker centromeric histone H3 and centrin localized at basal bodies (Additional file 5: Fig. S4). Interestingly, in the membrane fractions, the amount of GlPLK increased in the G2/M phase compared to that in the G1/S phase. However, our data cannot provide any evidence for the nuclear localization of GlPLK. The nuclear localization signal (NLS) and destruction box (D-box) were not observed in the amino acid sequence of GlPLK, whereas PLK1 has canonical sequences for the NLS and D-box [32]. Studies have shown that PLK1 SUMOylation is involved in its nuclear localization [33, 34]. A putative SUMO interaction sequence and a target sequence for SUMO were found in GlPLK using a SUMOylation prediction program (GPS SUMP 1.0). The absence of the D-box in cyclin B, AK and PLK of *G. lamblia* indicates a regulatory mechanism other than ubiquitin-mediated degradation [35]. Therefore, it will be interesting to study how the SUMOylation of GlPLK affects its localization and function during the cell cycle of *G. lamblia*.

In mammalian systems, PLK1 interacts with other proteins* via* its PBDs, and these interactions are critical for the spatial and temporal function of PLKs as they control their subcellular localization [36]. The roles of KD and PBD in the localization of GlPLK were examined using *Giardia* ectopically expressing truncated GlPLK proteins (Fig. 4). While the localization of GlPLK-PBD was similar to that of full-length GlPLK (Figs. 3c, 4d), the localization of GlPLK-KDL at mitotic spindles was not detected (Fig. 4c). Even though GlPLK-KDL was found together with GlCentrin at basal bodies, these double-stained basal bodies were misplaced in dividing cells, suggesting that GlPLK may be required for the proper function of basal bodies during cell division. Interestingly, the median bodies in trophozoites expressing truncated GlPLKs showed stronger labeling than those expressing full-length GlPLK.

When *Giardia* cells at different phases of the cell cycle were prepared by chemical treatment, both real-time PCR and western blotting showed upregulated GlPLK expression in cells in the G2/M phase compared with cells in the G1/S phase (Additional file 4: Fig. S3). An increased amount of phospho-GlPLK was also detected during the G2/M phase, as shown by western blotting using anti-phospho-PLK antibodies (Fig. 6a). In humans, the level of PLK1 is at its peak at metaphase [26]. A study using counterflow centrifugal elutriation of *Giardia* cells revealed a twofold increase in *glplk* gene expression in the G2/M phase [5].

GlPLK autophosphorylation has been demonstrated* in vitro* using rGlPLK synthesized* in vitro* (Fig. 7c). Mutagenesis of GlPLK and kinase assays using the mutant rGlPLKs confirmed that Lys51 is a critical residue that receives phosphate from ATP. Two putative phosphorylation residues, Thr179 and Thr183, play a complementary and redundant role, based on the observation that phosphorylation was dramatically affected only when both of the residues were mutated.

These situations are more complex* in vivo* because PLK1 phosphorylation can occur in spatial and temporal modes. This phosphorylation depends upon the correct localization to the site at which the target protein is present and on the binding of the target proteins to the PBD of PLK1 [37]. When mammalian PLK1 is phosphorylated by aurora kinase (AK) A, mitosis is initiated in the cells [38]. In addition, cyclin B-CDK1-dependent phosphorylation of aurora borealis is a prerequisite for PLK activation [39]. GlAK was found in basal bodies (in interphase and dividing cells) and mitotic spindles (in dividing cells), and AK inhibition resulted in a defect in cytokinesis [24, 25, 40]. These results suggested that GlPLK may function together with GlAK during the cell cycle of *G. lamblia*. Moreover, an interaction between these two kinases was observed* via* co-immunoprecipitation (Kim et al., unpublished results).

The role of GlPLK was further confirmed by ectopically expressing mutant GlPLK in *Giardia* trophozoites (Fig. 8b, c). In addition to cytokinesis, the expression of mutant GlPLK proteins (K51R and T179AT183A) inhibited the growth of *Giardia* trophozoites, indicating that GlPLK affects cell division. However, the expression of wild-type GlPLK mildly affected cell growth and did not exert any effect on cytokinesis in *G. lamblia*. This result demonstrated that the amino acid residues critical for GlPLK phosphorylation are also important for GlPLK function* in vivo*.

Lastly, we wish to address the effect of the GlPLK defect on flagellar homeostasis. While the cytoplasmic portion of the flagella was not affected, both GW-mediated and morpholino-mediated depletion of GlPLK resulted in the extension of the membrane-bound portion of the flagella in *Giardia* (Figs. 2, 5d)*.* Interestingly, the membrane-bound regions of the caudal and anterior flagella were dramatically extended, whereas those of the posterolateral flagella were not significantly affected. The differential effect of GlPLK defects on the four types of flagella may be derived from the pattern of the redistribution of the flagella during *Giardia* cell division in which the posterolateral and ventral flagella are inherited from mother cells, whereas the other two flagella are newly synthesized [41]. The role of GlPLK in the homeostasis of flagella formation could be implied from its localization pattern in *Giardia* trophozoites during interphase, wherein it shows localization to the flagella and axonemes (Fig. 3c).

Phosphorylation of GlPLK is essential for its function in regulating flagella length, as *Giardia* cells ectopically expressing mutant phosphorylation-negative GlPLK proteins also showed extended flagella (Fig. 8d). It is interesting that the localization of phospho-GlPLK was distinct in the cytoplasmic portion of the anterior flagella and flagella tips (Fig. 6b). Another labeled structure found in these IFAs is median bodies, whose function and biogenesis are not clear (Figs. 3c, d, 4c, d, 5b). However, the median body is postulated as a reservoir for MT and a regulator of MT homeostasis. Localization of GlPLK to basal bodies, which function as an MTOC, indicated that GlPLK might play a role in MT nucleation. A previous study demonstrated that the depletion of the γ-tubulin ring complex (γ-TuSC) affects MT nucleation, resulting in the shortening of the flagella [18]. Overexpression of dominant-negative mutant kinesin-13, a motor protein, resulted in *Giardia* with longer flagella and defective mitotic spindles [42]. Core machineries for flagella biogenesis and control in *Giardia* have been investigated, revealing the presence of intraflagellar transport (IFT)-mediated assembly [43]. The function of *G. lamblia* kinesin-13 at flagella pores results in the disassembly of flagella, as demonstrated by live-cell imaging and mathematical modeling of the conserved components of IFT and kinesin-13 [44]. IFT-mediated assembly and kinesin-mediated disassembly are key processes in controlling the length of the flagella or cilia in *Chlamydomonas* or *Tetrahynema thermophiles* [45, 46]. Our study added GlPLK to the list of components involved in the control of interphase flagellar length. In addition, flagella biogenesis has been reported to be an important factor for the cytokinesis of *Giardia* instead of actin-myosin-mediated mechanisms [6]. Interestingly, flagella shortening occurred in *Giardia* trophozoites in which expression of one of the 198 never-in-mitosis A (nimA)-related kinase (Nek) in *Giardia*, Nek8445, was depleted [47]. These studies suggest that GlPLK modulates flagella biogenesis* via* interaction with and/or modification of these proteins.

## Conclusions

In this study, we demonstrated that *G. lamblia* has one PLK, which functions in the cell cycle and in flagella formation, as revealed by inhibitor-mediated and morpholino-mediated inhibition. We also demonstrated that the phosphorylation of GlPLK plays an important role in cell growth, cytokinesis and flagella biogenesis in *Giardia*.

## Supplementary Information

**Additional file 1: Figure S1.** Effects of the PLK inhibitor GW843682X (*GW*) on *Giardia* growth. Growth inhibition of *G. lamblia* by GW. The numbers of *Giardia* trophozoites were counted using a hemocytometer 24-h post-treatment with various concentrations of GW (5–15 μM).

**Additional file 2: Table S1.** Percentages of cells with different nuclei number upon GW treatment.

**Additional file 3: Figure S2.** Sequence alignment of putative *G. lamblia* PLK amino acids (GL50803_104150) with those of *Trypanosoma brucei* (Tb927.7.6310) and human (NP_005021.2). Identical residues are indicated with asterisks, whereas homologous residues are represented with dots. The serine/threonine kinase domain at the amino-terminal is denoted by a shaded box. Two blocks of amino acids near the carboxyl terminus were proposed to be polo-box domains. The activation domain (T-loop) of the kinase domain is underlined. A lysine (*K*) in the amino-terminus region is suggested as a residue that initially receives phosphate from ATP, and two threonine residues in the T-loop are proposed as target sites of subsequent phosphorylation (indicated by bold letters).

**Additional file 4: Figure S3.** Expression of GlPLK in synchronized *Giardia* cells*.* **a** Flow cytometric analysis of *Giardia* trophozoites carrying pGlPLK.neo treated with 0.01% DMSO (interphase), trophozoites arrested with 100 nM nocodazole for 3 h (G2/M), and trophozoites treated with 100 nM nocodazole for 3 h, followed by incubation with 6 μM aphidicolin for 6 h (G1/S). **b** (**i**) Western blotting of synchronized cells using anti-HA antibodies. Lanes:* 1* *Giardia* carrying the empty vector,* 2*–*4* *Giardia* carrying pGlPLKHA.neo; lanes: * 2* interphase cells,* 3* G1/S-phase cells,* 4* G2/M-phase cells. The amount of GlPDI1 was also monitored in these cells using anti-GlPDI1 antibodies. (**ii**) Relative levels of GlPLK to GlPDI1 are expressed as a bar graph. **c** Real-time quantitative assay. The mRNA quantity in the cDNA samples was normalized using the *glactin* transcript levels. Data are presented as the average of three independent experiments. *P = 0.01–0.05.

**Additional file 5: Figure S4.** Subcellular fractionation of GlPLK in *G. lamblia*. *Giardia* carrying pGlPLK.neo was used to perform subcellular protein fractionation experiments. Both cytoplasmic and membrane protein fractions were prepared from interphase, G1/S-phase and G2/M-phase cells* via* sequential treatment with hypotonic and high-salt buffer. The amount of HA-tagged GlPLK in the extracts was monitored using anti-HA antibodies. The amount of GlGAP1, a cytoplasmic marker, was also detected using anti-GlGAP1 antibodies. On the other hand, GlCentrin was monitored as a marker protein for membrane fractions including basal bodies. As a marker for nuclear proteins, centromeric histone H3 was detected in these extracts using anti-GlCenH3 antibodies. Interphase cells, cytoplasmic fraction (*C*, lane 1), membrane fraction (*M*, lane 2); G1/S-phase cells: C (lane 3), M (lane 4); G2/M-phase cells: C (lane 5), M (lane 6).

## Data Availability

Data supporting the conclusions of this article are included within the article and its Additional files.

## Abbreviations

AK: Aurora kinase
CDK1: Cyclin-dependent kinase 1
GlCENH3: *Giardia lamblia* centromeric histion H3
GlCentrin: *Giardia lamblia* centrin
GlGAP1: *Giardia lamblia* glyceraldehyde 3-phosphate dehydrogenase
GlPLK1: *Giardia lamblia* PLK1
GW: PLK-specific inhibitor GW843286X
KD: N-terminal kinase domain
MT: Microtubules
MTOC: Microtubule-organizing center
PBD: Polo-box domain
PLK: Polo-like kinase
