# Supplementary figures and images for: A polo-like kinase modulates cytokinesis and flagella biogenesis in Giardia lamblia
Source: Parasit Vectors. 2021 Mar 31;14:182. doi: 10.1186/s13071-021-04687-5 (PMC8011197; doi:10.1186/s13071-021-04687-5)

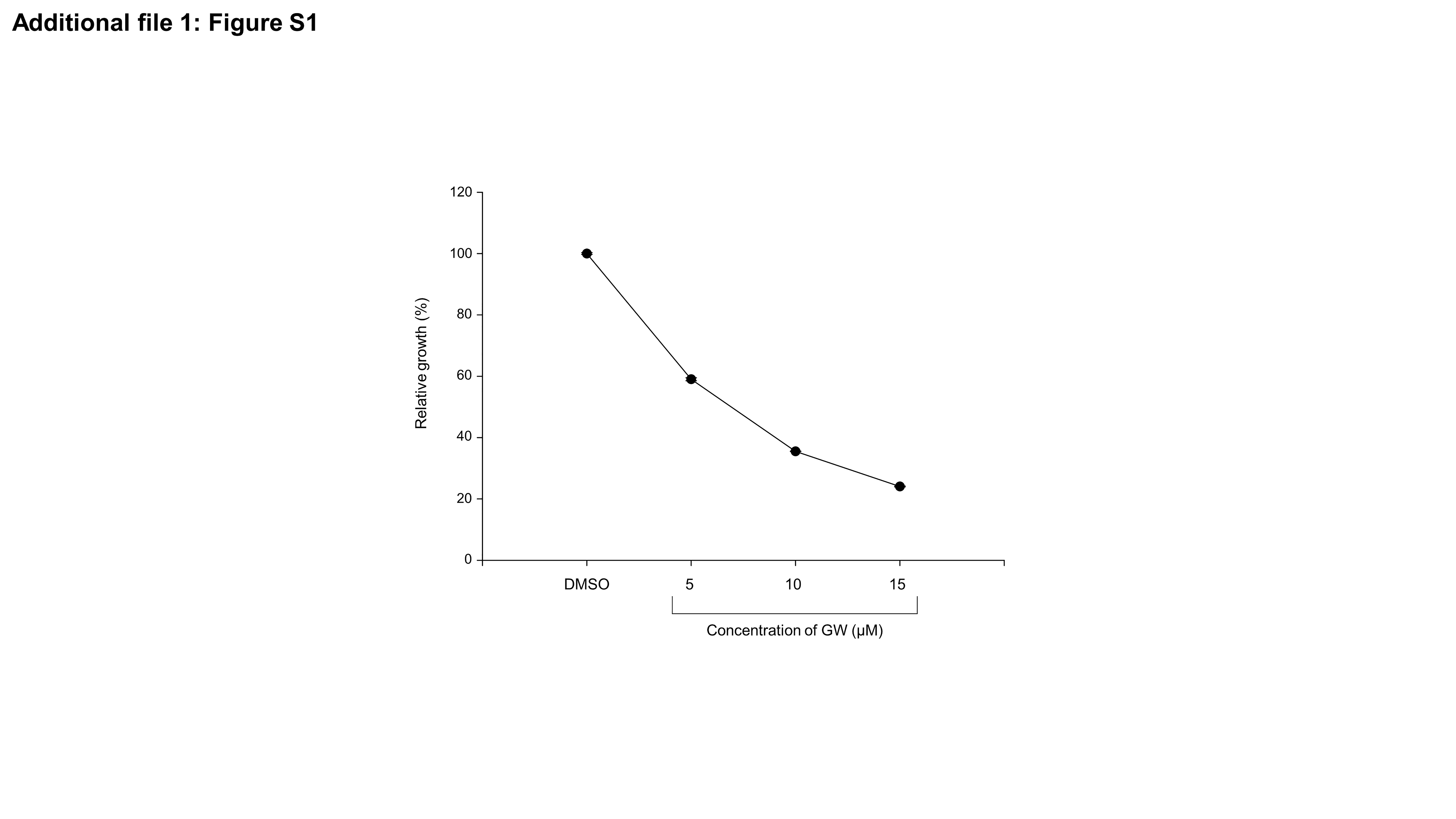

Supplement: Supplementary file 1 — Additional file 1: Figure S1. Effects of the PLK inhibitor GW843682X (GW) on Giardia growth. Growth inhibition of G. lamblia by GW. The numbers of Giardia trophozoites were counted using a hemocytometer 24-h post-treatment with various concentrations of GW (5–15 μM). [file 13071_2021_4687_MOESM1_ESM.tif]

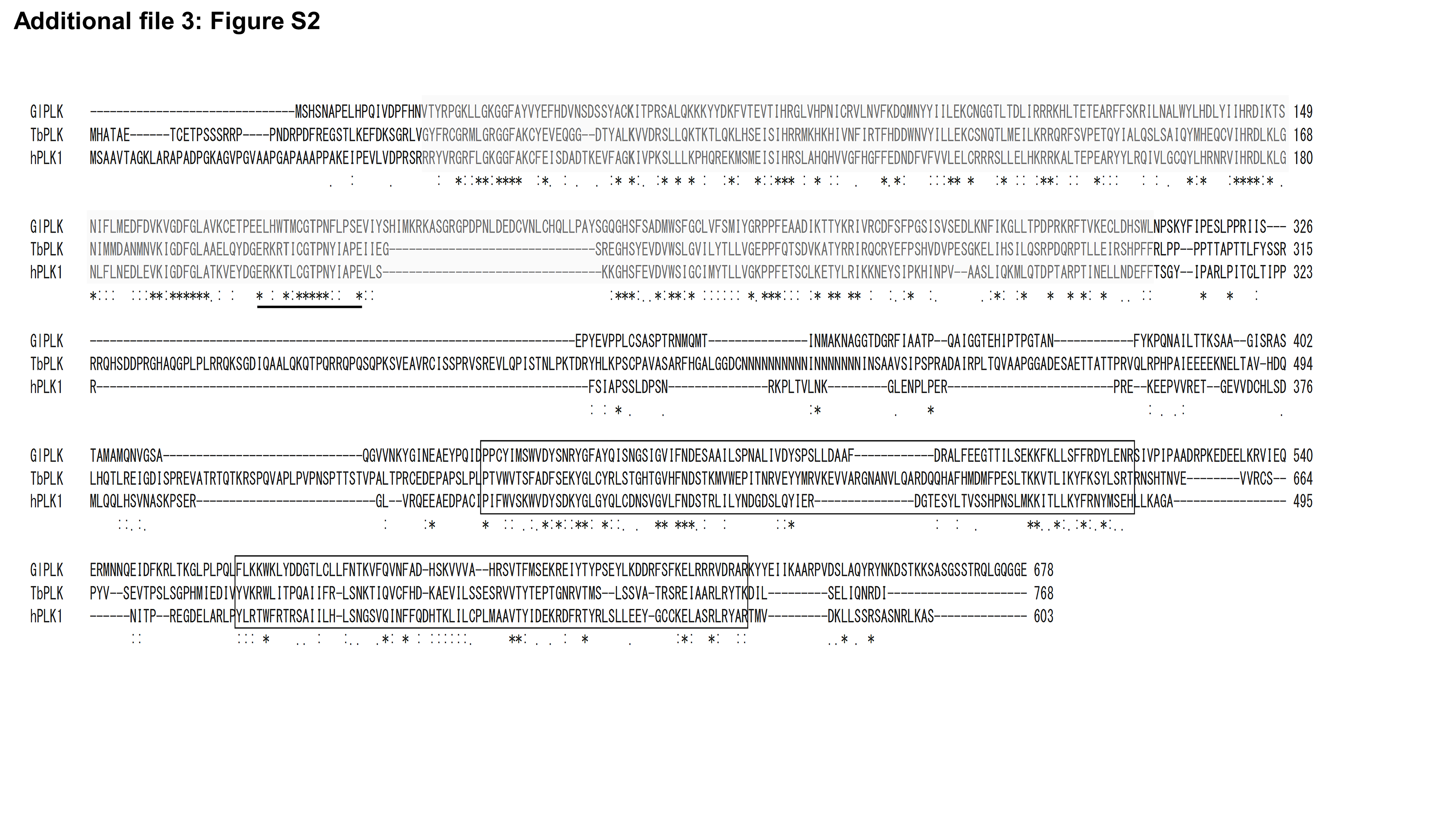

Supplement: Supplementary file 3 — Additional file 3: Figure S2. Sequence alignment of putative G. lamblia PLK amino acids (GL50803_104150) with those of Trypanosoma brucei (Tb927.7.6310) and human (NP_005021.2). Identical residues are indicated with asterisks, whereas homologous residues are represented with dots. The serine/threonine kinase domain at the amino-terminal is denoted by a shaded box. Two blocks of amino acids near the carboxyl terminus were proposed to be polo-box domains. The activation domain (T-loop) of the kinase domain is underlined. A lysine (K) in the amino-terminus region is suggested as a residue that initially receives phosphate from ATP, and two threonine residues in the T-loop are proposed as target sites of subsequent phosphorylation (indicated by bold letters). [file 13071_2021_4687_MOESM3_ESM.tif]

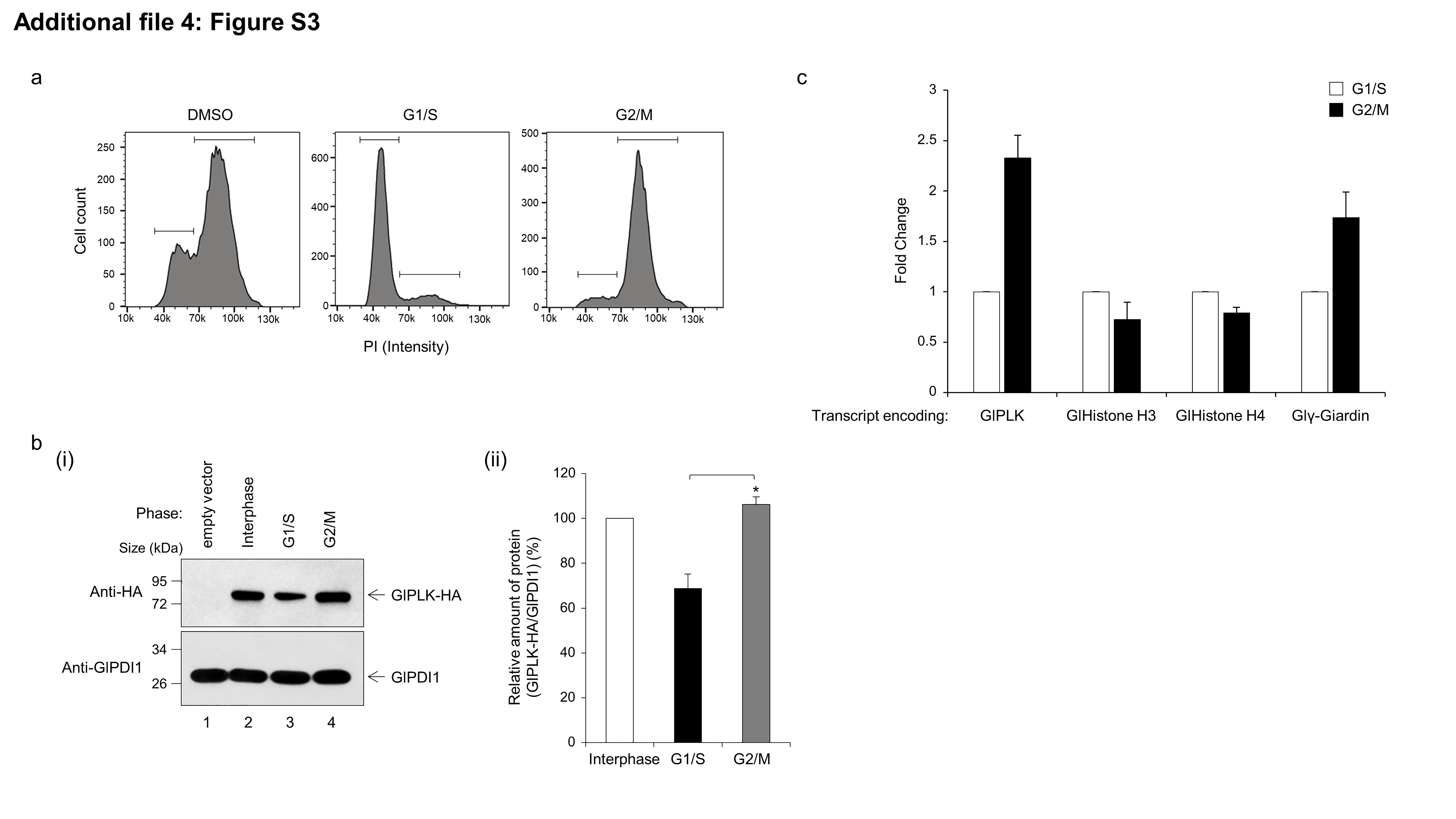

Supplement: Supplementary file 4 — Additional file 4: Figure S3. Expression of GlPLK in synchronized Giardia cells. a Flow cytometric analysis of Giardia trophozoites carrying pGlPLK.neo treated with 0.01% DMSO (interphase), trophozoites arrested with 100 nM nocodazole for 3 h (G2/M), and trophozoites treated with 100 nM nocodazole for 3 h, followed by incubation with 6 μM aphidicolin for 6 h (G1/S). b (i) Western blotting of synchronized cells using anti-HA antibodies. Lanes: 1 Giardia carrying the empty vector, 2–4 Giardia carrying pGlPLKHA.neo; lanes: 2 interphase cells, 3 G1/S-phase cells, 4 G2/M-phase cells. The amount of GlPDI1 was also monitored in these cells using anti-GlPDI1 antibodies. (ii) Relative levels of GlPLK to GlPDI1 are expressed as a bar graph. c Real-time quantitative assay. The mRNA quantity in the cDNA samples was normalized using the glactin transcript levels. Data are presented as the average of three independent experiments. *P = 0.01–0.05. [file 13071_2021_4687_MOESM4_ESM.tif]

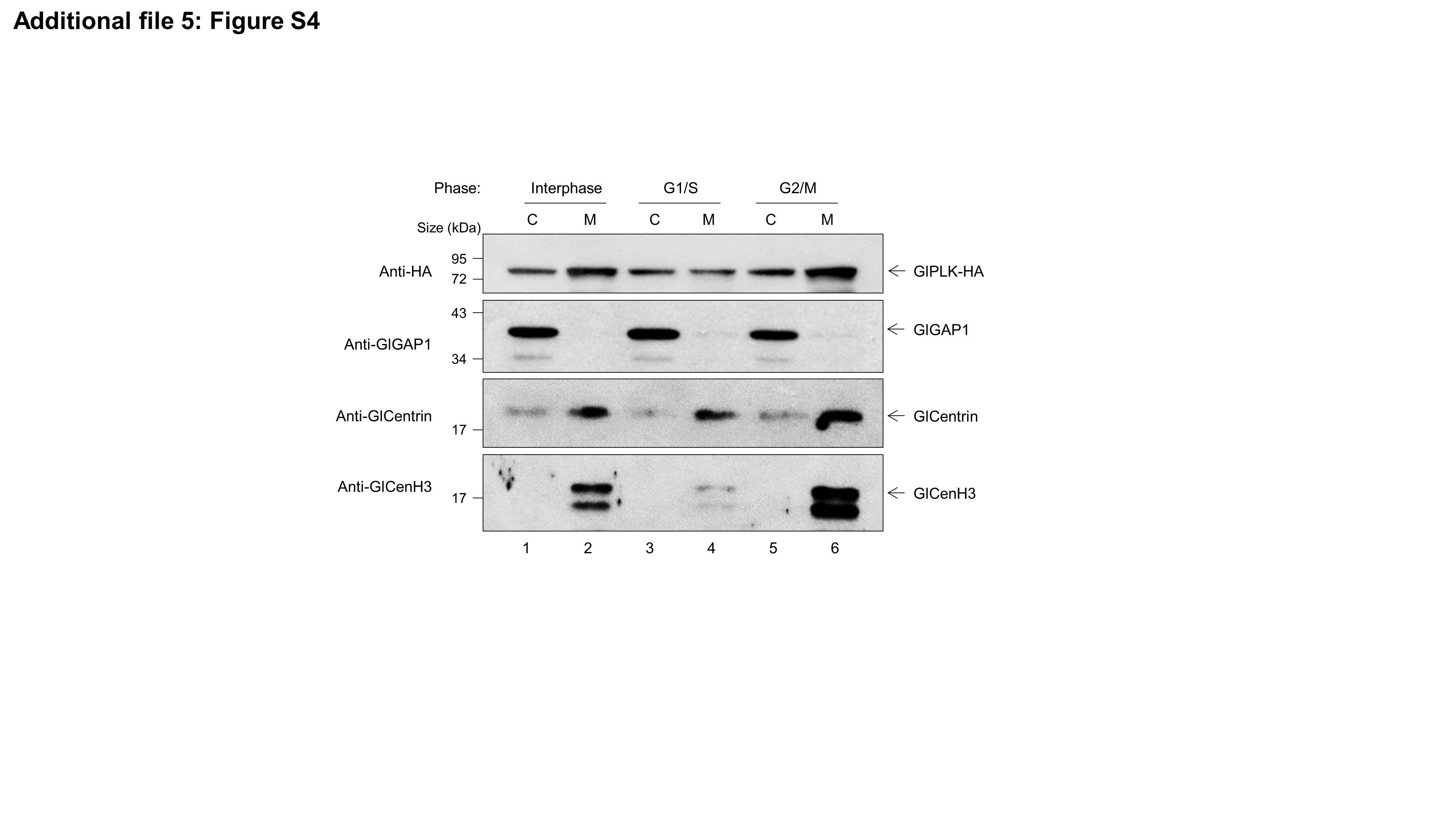

Supplement: Supplementary file 5 — Additional file 5: Figure S4. Subcellular fractionation of GlPLK in G. lamblia. Giardia carrying pGlPLK.neo was used to perform subcellular protein fractionation experiments. Both cytoplasmic and membrane protein fractions were prepared from interphase, G1/S-phase and G2/M-phase cells via sequential treatment with hypotonic and high-salt buffer. The amount of HA-tagged GlPLK in the extracts was monitored using anti-HA antibodies. The amount of GlGAP1, a cytoplasmic marker, was also detected using anti-GlGAP1 antibodies. On the other hand, GlCentrin was monitored as a marker protein for membrane fractions including basal bodies. As a marker for nuclear proteins, centromeric histone H3 was detected in these extracts using anti-GlCenH3 antibodies. Interphase cells, cytoplasmic fraction (C, lane 1), membrane fraction (M, lane 2); G1/S-phase cells: C (lane 3), M (lane 4); G2/M-phase cells: C (lane 5), M (lane 6). [file 13071_2021_4687_MOESM5_ESM.tif]
